# Clustering analysis of large-scale phenotypic data in the model filamentous fungus *Neurospora crassa*

**DOI:** 10.1186/s12864-020-07131-7

**Published:** 2020-11-02

**Authors:** Alexander J. Carrillo, Ilva E. Cabrera, Marko J. Spasojevic, Patrick Schacht, Jason E. Stajich, Katherine A. Borkovich

**Affiliations:** 1 Department of Microbiology and Plant Pathology, University of California, 900 University Avenue, Riverside, CA 92521 USA; 2 Department of Evolution, Ecology, and Organismal Biology, University of California, Riverside, California 92521 USA

**Keywords:** Functional genomics, Hierarchical clustering, Fungal genetics, Neurospora, Phenotypic analysis

## Abstract

**Background:**

With 9730 protein-coding genes and a nearly complete gene knockout strain collection, *Neurospora crassa* is a major model organism for filamentous fungi. Despite this abundance of information, the phenotypes of these gene knockout mutants have not been categorized to determine whether there are broad correlations between phenotype and any genetic features.

**Results:**

Here, we analyze data for 10 different growth or developmental phenotypes that have been obtained for 1168 *N. crassa* knockout mutants. Of these mutants, 265 (23%) are in the normal range, while 903 (77%) possess at least one mutant phenotype. With the exception of unclassified functions, the distribution of functional categories for genes in the mutant dataset mirrors that of the *N. crassa* genome. In contrast, most genes do not possess a yeast ortholog, suggesting that our analysis will reveal functions that are not conserved in *Saccharomyces cerevisiae*. To leverage the phenotypic data to identify pathways, we used weighted Partitioning Around Medoids (PAM) approach with 40 clusters. We found that genes encoding metabolic, transmembrane and protein phosphorylation-related genes are concentrated in subsets of clusters. Results from K-Means clustering of transcriptomic datasets showed that most phenotypic clusters contain multiple expression profiles, suggesting that co-expression is not generally observed for genes with shared phenotypes. Analysis of yeast orthologs of genes that co-clustered in MAPK signaling cascades revealed potential networks of interacting proteins in *N. crassa*.

**Conclusions:**

Our results demonstrate that clustering analysis of phenotypes is a promising tool for generating new hypotheses regarding involvement of genes in cellular pathways in *N. crassa*. Furthermore, information about gene clusters identified in *N. crassa* should be applicable to other filamentous fungi, including saprobes and pathogens.

## Background

*Neurospora crassa* is a model organism for studies of cellular biology and genetics in filamentous fungi [1, 2]. Filamentous fungi can be pathogens of plants and animals, but also form beneficial endophytic associations with plants (reviewed in [3–5]). Many filamentous fungi are crucial players during carbon cycling in the environment and serve as commercial sources of food, drink, biofuels and other products [6–8]. The contributions of *N. crassa* to many areas of cell and molecular biology include the one-gene, one-polypeptide hypothesis, genetic recombination, gene silencing by small RNAs, epigenetic phenomena, photobiology, circadian rhythms, cell signaling, plant cell wall decomposition, and self-nonself interactions during vegetative growth and sexual development (reviewed in [1, 2]).

*N. crassa* has a rich history of forward genetics, with more than 1000 loci identified (http://www.fgsc.net/2000compendium/2000compend.html) [9]. *N. crassa* was the first filamentous fungus with a complete genome sequence [10]. The ~ 40 Mb genome contains ~ 10,000 protein-coding genes distributed among seven linkage groups [10]. One goal of the Neurospora Genome project was to delete all of the genes in the *N. crassa* genome using reverse genetics [11, 12]. The project produced knockout mutant strains for nearly 9000 genes and these mutants are currently available at the Fungal Genetics Stock Center [13]. In all knockout mutants, a gene open reading frame has been replaced with an *hph* selectable marker (conferring resistance to the antibiotic hygromycin) [11, 14]. Another goal of the Neurospora Genome Project was to perform phenotypic characterization of these knockout mutants. An undergraduate research program at the University of California, Los Angeles, pioneered methods for phenotyping mutants, with ~ 1000 mutants analyzed [15]. In our laboratory, we have used the phenotypic methods developed by the UCLA project to analyze additional mutants, focusing on those lacking serine/threonine protein kinases, serine/threonine and tyrosine protein phosphatases, G protein coupled receptors and transcription factors [16–19].

The above projects have generated phenotypic data for nearly 1300 *N. crassa* knockout mutants. However, these data have not been otherwise analyzed or interpreted using hierarchical/partitional statistical methods. Such approaches have been used in some fungal species, where phenomics was used to predict relationships between genes [20–22]. All of these studies utilized either only quantitative data or converted categorical phenotypes to a numeric scale followed by generation of a distance matrix and application of Pearson’s correlation [23] to perform further analysis and interpretation. However, there are no published reports of analysis of gene deletion mutants in fungi where phenotypes were clustered without conversion of categorical data to an arbitrary numerical value, nor studies that include purely categorical data in clustering analyses.

In this study, we curated phenotypic data for 10 categorical or quantitative/continuous traits for 1168 *N. crassa* knockout mutants obtained from the above projects. We then clustered the data without conversion to arbitrary values using Partitioning Around Medoids (PAM) [24]. Further, transcriptomics data from three publicly available datasets were clustered using K-means and the resulting expression profiles compared to the phenotypic clusters to determine whether gene expression correlated with phenotype. Our results reveal previously unknown relationships between phenotypes and genes encoding proteins with particular domains and between phenotypes and gene expression trends. These results have led to new hypotheses regarding cellular pathways that can be the subject of follow-up studies.

## Results

### *N. crassa* mutant defects are distributed across broad growth and developmental phenotypes

This dataset contains phenotypes for 379 mutants previously reported in five publications [11, 16–19], corresponding to 242 transcription factor, 36 GPCR, 24 serine-threonine-tyrosine protein phosphatase and 77 serine-threonine protein kinase gene mutants. The dataset also includes phenotypes for 789 mutants that were not previously published. Phenotypic data for all mutants is available in Additional file 1 and at the FungiDB database on the specific gene’s page.

**Fig. 1:**
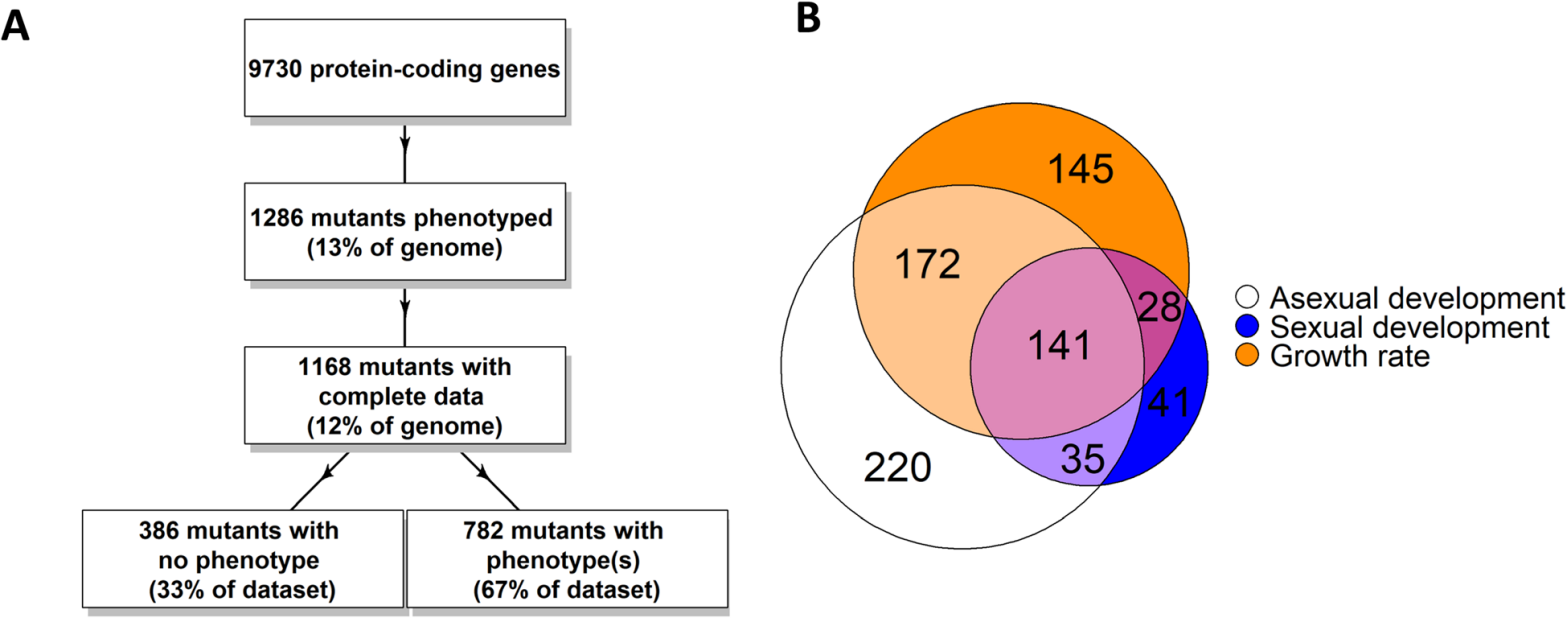
Summary of results from phenotypic analysis of 1286 *N. crassa* gene replacement mutants. **a**. Flow chart for phenotypic analysis. The number and features of mutants analyzed at each step of the process are noted. The percentage of “No phenotype” and “With phenotype(s)” mutants refers to strains with complete data. **b**. Major phenotypic classes of mutants. The distribution of mutants with phenotypes in at least one of the three major categories (growth rate, asexual development and sexual development) is shown in the lobes of the Venn diagram. The intersection of lobes indicates mutants with defects in more than one major category

During curation, we settled on 10 traits that were easiest for students to score and were therefore the most reliable. These include hyphal growth rate on solid medium, aerial hyphae height, conidia amount and morphology, abundance and appearance of unfertilized and fertilized female reproductive structures (protoperithecia and perithecia) and sexual spores (ascospores). Since the last eight phenotypes were scored using visual screens, amount/abundance is semi-quantitative (see Methods). In all, 1286 knockout mutants had phenotypic data for at least one trait (13% of genome) and 1168 (12% of genome) had complete data for the 10 traits included in our analysis (Fig. 1a; Additional file 1). Of the 1168 mutants with complete data, 903 mutants (77%) possessed at least one defect. We grouped the phenotypes for the 903 mutants with at least one defect into three global phenotypic classes: growth rate, asexual development (conidiation or aerial hyphae) and sexual development (protoperithecia, perithecia and ascospores; Fig. 1b). This yielded 1539 total global phenotypes for the 903 mutants. A total of 742 mutants (48.2%) had growth rate defects, 553 (35.9%) possessed an asexual developmental defect and 244 (15.8%) had a phenotype during sexual development. The lower overall incidence of sexual cycle phenotypes was previously noted in published results for GPCRs and transcription factors, and may reflect the large number of mutants in these two classes (36 and 242 mutants, respectively) in our combined dataset.

### The genes in the phenotypic dataset broadly mirror those of the *N. crassa* genome

**Fig. 2:**
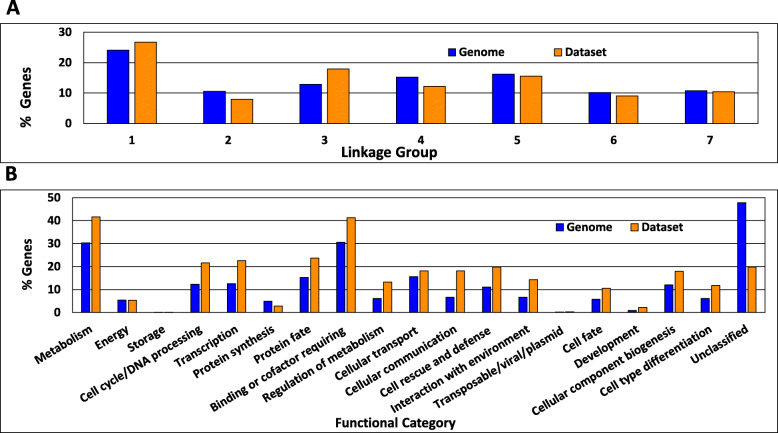
Distribution of genes by linkage group and functional catalogue assignments. **a**. Genes on different linkage groups. The distribution of all *N. crassa* genes on different linkage groups and the genes in each linkage group deleted in knockout mutants with complete phenotypic data are shown. Raw data are available in Additional file 1. **b**. Genes by functional catalogue (FunCat) assignment. The distribution of FunCat assignments for all genes in the *N. crassa* genome and the genes deleted in knockout mutants with complete phenotypic data is shown. Functional category data are available at https://elbe.hki-jena.de/fungifun/fungifun.php

We next explored the similarity between our dataset and the *N. crassa* protein-coding genome at a global scale, for representation of genes on each of the seven chromosomes and in gene functional categories. We used a chi-square test to determine whether the distribution of genes in our dataset across all chromosomes is similar to that of the entire genome. The low *p*-value (0.0000038) formally rejects goodness of fit of the data in Fig. 2a, and, therefore, there is no strong correlation between the dataset genes and the genome. This is corroborated by the observation that our dataset is overrepresented by approximately 5–10% on chromosomes 1 and 3, and underrepresented by 2–3% on chromosomes 2 and 4, while the distribution on the other three chromosomes was within 1% of the genome (Fig. 2a).

We investigated the distribution of functions of the mutated genes in our dataset vs. all genes in the genome using Functional Catalogue (FunCat) analysis with data from FungiFun2 (Fig. 2b). With the exception of protein synthesis and unclassified proteins, our dataset was overrepresented in each category by 2–10%, with the average overrepresentation approximately 6.2%. Taking into account all categories over- and underrepresented, the average difference from the genome is +/− 3.7%. Thus, the findings from analyzing the distribution of genes on chromosomes and their functional categories indicate that our phenotypic dataset is broadly representative of the *N. crassa* genome.

### Analysis using partitioning around Medoids (PAM) yielded the most biologically relevant phenotypic clusters

As there are many challenges associated with clustering mixed data (Floss et al. 2018), we initially tested several algorithms for hierarchical and partitioning clustering in order to uncover new biological pathways, including Pearson’s Correlation Coefficient [23], K-means [25], Factor Analysis of Mixed Data (FAMD) [26], Ward’s minimum variance (Ward’s) [27], Partitioning Around Medoids (PAM) [24] and K-prototypes [28]. Analysis scripts used in this study are available in Additional file 2.

We created guidelines that we could use to compare each method against each other to determine the most biologically relevant clustering. We first set a lower limit of three genes per cluster with a maximum of 40 clusters (Additional file 3). We next calculated the average relative standard deviation for basal hyphae growth rate and aerial hyphae height for each cluster and then averaged across all clusters (Additional file 4). For the categorical traits, we determined the most prevalent category for each cluster and determined an average across all clusters. These metrics allowed us to set guidelines for these continuous variables (Additional file 4). We set a limit of less than 15% relative standard deviation and no less than 95% average consensus for the clustering.

We first tried methods that required a conversion of categorical data into numerical values. The two methods, Pearson’s Correlation Coefficient [23] and K-means [25], provided relative standard deviations for basal hyphae growth rate and aerial hyphae height above the 15% cutoff (Additional file 1, Additional file 4). Based on the unsatisfactory results using the converted dataset, we turned to methods that would require little or no pre-processing of our data and that would retain the categorical data. One algorithm that can handle such mixed data is FAMD [26]. However, this approach quickly failed our criteria, as the run with three total clusters contained one cluster with a single gene (Additional file 3). Additionally, K-Prototypes (10.1023/A:1009769707641) was found to be unstable and multiple runs would not converge on similar numbers of clusters. We next utilized the Daisy function [29] and Gower’s metric [30] from the “cluster” R package to create a dissimilarity matrix (r-project.org). We then used Ward’s [24] or PAM [27] algorithms to generate clusters. Both methods did not initially meet our criteria (Table 1, Additional file 4). However, with unequal weighting of each variable we found that these two methods could meet our criteria (Table 1, Additional file 4). Overall, we found that PAM weight six with 40 clusters had the best combination of lowest relative standard error and highest percent consensus (Table 1). A more detailed description of the results obtained using the various clustering methods is available in Additional file 5.

**Table 1:** Comparison of results using different iterations of Partitioning Around Medoids (PAM)

Weight^**c**^	Number of Clusters	Average % consensus^**a**^									Average relative standard deviation (RST)^**b**^		
		Conidia number	Conidia morphology	Protoperithecia number	Protoperithecia morphology	Perithecia number	Perithecia morphology	Ascospore number	Ascospore morphology	Average % consensus overall	Basal hyphae growth rate	Aerial hyphae height	Average RST overall
0	18	91.72	94.20	93.74	94.23	95.27	93.34	96.35	98.32	95.06	26.76	34.12	13.20
0	19	92.16	94.50	94.05	94.51	95.45	93.66	96.48	98.39	95.29	25.33	32.54	12.76
0	20	92.50	94.78	94.35	94.79	95.68	93.92	96.66	98.47	95.52	24.62	30.43	12.36
0	21	93.02	95.19	94.61	95.04	95.88	94.21	96.81	97.03	95.54	28.30	31.30	13.09
1	31	95.92	96.57	93.46	94.99	95.70	95.39	97.32	98.76	96.03	19.80	24.13	11.02
1	32	95.22	96.66	94.40	95.98	95.84	95.53	97.40	98.80	96.37	20.18	24.55	10.44
1	33	95.67	97.05	94.57	96.11	95.97	95.67	97.47	97.95	96.40	22.57	25.24	10.92
2	32	96.23	94.89	94.02	95.04	94.22	95.51	98.76	98.82	95.90	17.52	26.50	10.88
2	33	96.35	95.05	94.20	95.19	94.31	95.64	98.79	98.86	96.01	16.91	25.83	10.67
2	34	95.45	95.24	94.99	96.82	94.47	95.77	98.82	98.89	96.43	17.55	26.30	10.22
2	35	95.15	94.62	95.00	96.91	93.95	94.98	98.03	98.09	95.94	18.98	25.30	11.19
3	29	96.20	96.09	93.25	94.20	92.63	95.26	95.53	97.62	94.94	13.77	27.67	10.82
3	30	96.37	96.22	93.47	94.39	92.88	95.34	95.67	97.70	95.10	13.43	26.61	10.60
3	31	97.45	97.64	91.71	94.52	94.39	94.05	95.14	96.63	94.87	15.60	27.08	10.98
3	32	97.45	97.29	92.26	94.54	93.62	93.38	95.46	96.91	94.78	15.46	27.69	11.41
3	33	96.92	96.53	92.41	94.65	93.28	92.81	95.29	96.69	94.52	17.58	26.11	12.14
4	32	90.89	94.99	93.99	95.11	94.39	94.23	94.57	97.20	94.93	14.23	18.81	11.42
4	33	90.50	94.98	93.99	94.80	94.13	93.80	94.40	97.37	94.78	14.21	19.08	11.96
4	34	90.78	94.78	94.10	94.83	94.76	94.13	94.97	97.46	95.01	14.10	20.18	11.75
4	35	90.82	94.93	94.27	94.98	94.91	94.23	95.11	97.53	95.14	13.78	19.68	11.57
5	36	85.84	95.66	94.14	95.40	95.38	93.87	94.33	97.42	95.17	14.09	13.44	11.26
5	37	85.28	95.45	94.18	95.41	95.51	94.04	94.48	97.49	95.22	13.99	12.45	11.22
5	38	85.18	95.55	94.88	95.63	94.53	94.15	94.03	97.55	95.19	13.90	12.05	11.20
5	39	84.56	95.62	95.24	96.70	94.67	94.30	94.18	97.61	95.48	13.93	12.46	11.00
5	40	84.36	95.37	96.40	96.26	94.37	94.01	94.33	97.67	95.49	13.80	12.71	10.96
6	39	89.07	95.73	94.33	94.58	93.57	93.70	93.89	97.39	94.03	14.11	11.64	11.03
6	40	89.64	95.83	94.22	94.71	93.73	93.86	94.04	97.45	94.19	13.94	11.86	10.98

^a^Average percent consensus - For each cluster the category that was most prevalent was determined and represented as a percent. Then each cluster’s most prevalent category was used to create a trait average

^b^Average relative standard deviation - For continuous variables, the standard deviation for each cluster was calculated and then divided by the cluster mean to determine the relative standard deviation

^c^Weight - Each phenotype was assigned a specific weight relative to the other traits when creating the Gower’s distance matrix. The phenotypes are in the order as shown in Table 1

No weight = (1,1,1,1,1,1,1,1,1,1); Weight 1 = (1,1,1,1,1,1,1,1,2,2); Weight 2 = (1,0.5,1,0.5,1,0.5,1,0.5,2,2); Weight 3 = (2,2,1,1,1,1,1,1,6,2); Weight 4 = (0.5,0.5,1,1,1,1,1,1,6,5); Weight 5 = (1,1,1,1,1,1,1,1,6,4); Weight 6 = (1,1,1,1,1,1,1,1,6,6)

**Fig. 3:**
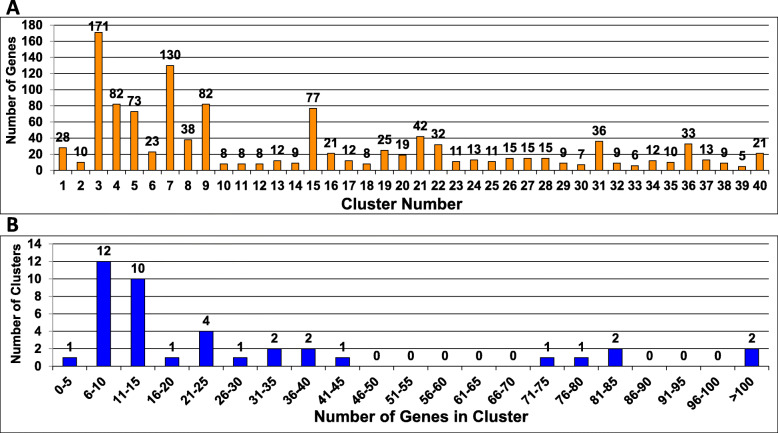
Grouping of mutants in clusters. **a**. Number of genes per cluster. The number of mutants in each cluster is shown. Data are taken from Additional file 1. The clustering algorithm was a weighted PAM. See Methods for details. The numbers above the bars indicate the number of genes per cluster. **b**. Number of clusters in different size ranges. Clusters were sorted into bins according to the number of genes in the cluster. The number of clusters corresponding to each size range (bin) is shown. The numbers above the bars indicates the number of clusters in each size range. Raw data are available in Additional file 1

The weighted PAM 40 method effectively separated mutants into distinct clusters, without formation of different clusters with identical phenotypes (Table 2). The average number of genes in each cluster was 29.2, and the median size was 14, with a range from 5 (Cluster 39) to 171 (Cluster 3) genes (Additional file 1, Fig. 3a). Most clusters contained 6–15 genes (Fig. 3b). We subsequently analyzed the Weighted PAM 40 clusters for the presence of different classes of genes or other attributes, as described in the following sections.

**Table 2:** Phenotype summary for 40 PAM clusters

Cluster #	# Genes in cluster	% Mutants with same phenotype^**a**^									
		Basal hyphae growth rate	Aerial hyphae height	Conidial #	Conidial morph-ology	Protoperi-thecial #	Protoperi-thecial morph-ology	Peri-thecial #	Peri-thecial morph-ology	Asco-spore #	Asco-spore morph-ology
**1**	28	75% Normal Average	57% Normal High	93% Normal	100% Normal	100% Normal	100% Normal	100% Normal	100% Normal	100% Normal	100% Normal
**2**	10	90% Reduced	80% Reduced	100% Reduced	100% Normal	100% Not Formed	100% Not Formed	100% Not Formed	100% Not Formed	100% Not Formed	100% Not Formed
**3**	171	94% Normal Low	95% Normal Average	99% Normal	100% Normal	100% Normal	100% Normal	100% Normal	99% Normal	100% Normal	100% Normal
**4**	82	88% Normal Average	85% Normal Average	98% Normal	100% Normal	100% Normal	100% Normal	100% Normal	100% Normal	100% Normal	100% Normal
**5**	73	100% Slightly Reduced	93% Reduced	100% Normal	100% Normal	99% Normal	100% Normal	100% Normal	100% Normal	100% Normal	100% Normal
**6**	23	100% Reduced	96% Reduced	100% Normal	100% Normal	96% Normal	100% Normal	100% Normal	100% Normal	100% Normal	100% Normal
**7**	130	100% Slightly Reduced	96% Normal Average	99% Normal	100% Normal	100% Normal	100% Normal	100% Normal	99% Normal	99% Normal	100% Normal
**8**	38	100% Reduced	100% Reduced	97% Normal	97% Normal	97% Normal	100% Normal	97% Normal	100% Normal	100% Normal	100% Normal
**9**	82	80% Normal Low	95% Reduced	100% Normal	100% Normal	100% Normal	99% Normal	99% Normal	100% Normal	95% Normal	100% Normal
**10**	8	62% Severely Reduced	75% Normal Average	75% Normal	100% Normal	88% Normal	100% Normal	62% Normal	88% Normal	75% Normal	100% Normal
**11**	8	50% Slightly Reduced	88% Reduced	100% Normal	100% Normal	100% Normal	75% Normal	88% Not Formed	88% Not Formed	100% Not Formed	100% Not Formed
**12**	8	88% Reduced	88% Reduced	100% Reduced	88% Normal	62% Normal	50% Normal	88% Not Formed	88% Not Formed	100% Not Formed	100% Not Formed
**13**	12	50% Reduced	58% Reduced	75% Reduced	92% Normal	92% Reduced	100% Normal	92% Reduced	92% Normal	83% Reduced	83% Normal
**14**	9	Varied	89% Normal Average	89% Normal	100% Normal	89% Normal	100% Normal	67% Normal	78% Normal	100% Not Formed	100% Not Formed
**15**	77	100% Slightly Reduced	96% Normal Average	100% Normal	100% Normal	100% Normal	100% Normal	97% Normal	100% Normal	99% Normal	100% Normal
**16**	21	Varied	67% Normal Average	95% Normal	95% Normal	86% Normal	90% Normal	100% Not Formed	100% Not Formed	100% Not Formed	100% Not Formed
**17**	12	100% Severely Reduced	92% Reduced	92% Normal	83% Normal	92% Normal	100% Normal	100% Normal	92% Normal	92% Normal	92% Normal
**18**	8	100% Severely Reduced	88% Reduced	75% Normal	88% Normal	88% Not Formed	88% Not Formed	100% Not Formed	100% Not Formed	100% Not Formed	100% Not Formed
**19**	25	52% Reduced	100% Increased	96% Normal	100% Normal	100% Normal	100% Normal	100% Normal	100% Normal	96% Normal	100% Normal
**20**	19	74% Severely Reduced	100% Reduced	84% Normal	95% Normal	95% Normal	95% Normal	100% Normal	100% Normal	89% Normal	100% Normal
**21**	42	100% Slightly Reduced	100% Reduced	100% Normal	100% Normal	100% Normal	98% Normal	100% Normal	100% Normal	100% Normal	100% Normal
**22**	32	100% Reduced	84% Normal Average	97% Normal	100% Normal	97% Normal	100% Normal	97% Normal	100% Normal	97% Normal	97% Normal
**23**	11	82% Reduced	91% Reduced	91% Normal	100% Normal	100% Not Formed	100% Not Formed	100% Not Formed	100% Not Formed	100% Not Formed	100% Not Formed
**24**	13	85% Reduced	85% Reduced	92% Normal	92% Normal	92% Normal	62% Normal	100% Not Formed	100% Not Formed	100% Not Formed	100% Not Formed
**25**	11	91% Reduced	100% Severely Reduced	55% Reduced	100% Normal	100% Not Formed	100% Not Formed	100% Not Formed	100% Not Formed	100% Not Formed	100% Not Formed
**26**	15	67% Slightly Reduced	100% Severely Reduced	100% Normal	100% Normal	100% Normal	100% Normal	100% Normal	100% Normal	97% Normal	100% Normal
**27**	15	Varied	60% Normal Average	100% Normal	100% Normal	80% Normal	100% Normal	87% Normal	87% Normal	93% Reduced	100% Normal
**28**	15	67% Reduced	100% Increased	80% Normal	100% Normal	93% Normal	93% Normal	100% Normal	93% Normal	87% Normal	100% Normal
**29**	9	67% Slightly Reduced	78% Reduced	89% Normal	100% Normal	89% Normal	89% Normal	78% Reduced	89% Normal	100% Not Formed	100% Not Formed
**30**	7	100% Severely Reduced	100% Severely Reduced	Varied	71% Normal	100% Normal	100% Normal	57% Normal	86% Normal	Varied	Varied
**31**	36	100% Reduced	92% Normal Average	97% Normal	100% Normal	100% Normal	97% Normal	94% Normal	100% Normal	94% Normal	100% Normal
**32**	9	56% Slightly Reduced	89% Reduced	100% Reduced	100% Normal	89% Normal	100% Normal	100% Normal	100% Normal	100% Normal	100% Normal
**33**	6	50% Reduced, 50% Severely Reduced	67% Normal Average	67% Normal	67% Normal	83% Reduced	100% Normal	83% Reduced	67% Abnorm-al Beaks	83% Not Formed	83% Not Formed
**34**	12	67% Reduced	83% Normal Average	83% Normal	75% Normal	92% Not Formed	92% Not Formed	100% Not Formed	100% Not Formed	100% Not Formed	100% Not Formed
**35**	10	60% Slightly Reduced	50% Normal Average	100% Normal	100% Normal	100% Reduced	90% Normal	90% Reduced	100% Normal	60% Normal	100% Normal
**36**	33	Varied	100% Increased	88% Normal	100% Normal	100% Normal	100% Normal	100% Normal	94% Normal	100% Normal	100% Normal
**37**	13	69% Reduced	85% Reduced	92% Normal	100% Normal	92% Normal	92% Normal	85% Normal	77% Normal	100% Reduced	100% Normal
**38**	9	67% Reduced	100% Severely Reduced	89% Normal	100% Normal	89% Normal	89% Normal	89% Normal	100% Normal	89% Normal	100% Normal
**39**	5	80% Reduced	60% Reduced	80% Normal	100% Normal	100% Normal	100% Normal	100% Normal	Varied	100% Not Formed	100% Not Formed
**40**	21	100% Severely Reduced	100% Severely Reduced	76% Reduced	90% Normal	90% Not Formed	90% Not Formed	100% Not Formed	100% Not Formed	100% Not Formed	100% Not Formed

^a^The value in the cell is the percentage of mutants with the indicated majority phenotype. Varied indicates that no one phenotype was present in more than 50% of the mutants. Normal range for growth rate is 75–85 mm/day. 75–77.5 = Normal Low; 77.6–82.5 = Normal Average; 82.6–85 = Normal High; 65–74.9 = Slightly Reduced; 40–64.9 = Reduced; < 40 = Severely Reduced; > 85 = Increased. Normal range for aerial hyphae height is 30-45 mm. 35.1–39.9 = Normal Average; 30–35 = Normal Low; 40–45 = Normal High; 25–29 = Slightly Reduced; 15–24.9 = Reduced; < 15 = Severely Reduced; > 45 = Increased

### The majority of cluster genes lack *Saccharomyces cerevisiae* orthologs

**Fig. 4:**
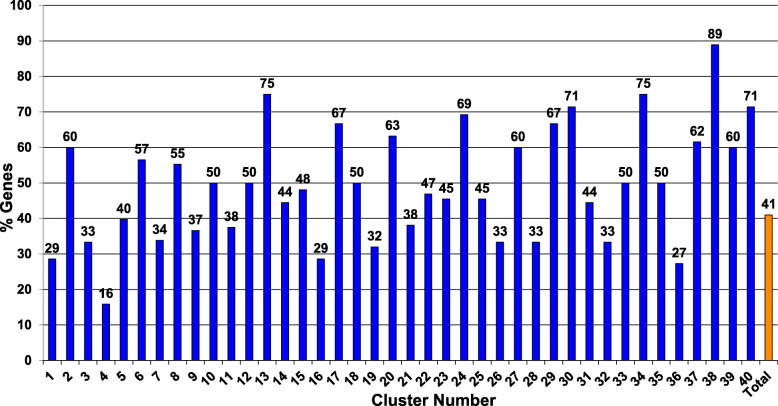
Placement of genes with yeast orthologs in clusters. The genes deleted in the mutants were analyzed for *S. cerevisiae* orthologs using the “Transform by Orthology” tool at the FungiDB database. The percentage of genes with orthologs in each cluster (blue bars), and in the entire dataset (orange bar), are shown. The numbers above the bars indicate the percent of genes in a cluster with a yeast ortholog. Raw data are available in Additional file 6

We cataloged which of the 9730 predicted genes in the *N. crassa* genome have orthologs in the model yeast *S. cerevisiae* using tools (Orthology Phylogenetic Profile) at the FungiDB database. The results showed that 3167 *N. crassa* genes (32.5% of the total) have an ortholog in *S. cerevisiae* (data not shown), but of the 1168 genes represented by knockout mutants in this study, 479 (41%) have a yeast ortholog (Fig. 4). The higher percentage of genes with orthologs in the knockout set likely reflects the results observed for FunCat analysis (Fig. 2), where genes with suspected functions were overrepresented in the phenotypic dataset.

We next determined the percentage of *N. crassa* genes in each of the 40 clusters that have yeast orthologs (Fig. 4; Additional file 6). The range was 16% (13 genes out of 81 total) in Cluster 4 to 89% in Cluster 38 (8 genes out of 9 total) (Fig. 4). In most clusters, a majority of genes lacked a yeast ortholog and in 21 clusters less than 50% of the genes possess yeast orthologs. Of note, the clusters with the highest proportion of yeast orthologs have reduced growth rates relative to wild type (Clusters 13, 30, 34, 38, and 40; Table 2), while those with the fewest yeast orthologs typically have normal or variable growth rate phenotypes (Clusters 1, 4, 16 and 36; Table 2). The relative lack of shared phenotypes among clusters with a low percentage of orthologous yeast genes may reflect specialized functions that evolved within filamentous fungi and since the divergence from a common ancestor at the origin of the ascomycetes.

Two of the clusters containing the highest proportion of genes with yeast orthologs are Clusters 34 (9 genes of 12 total; 75%) and 40 (15 genes of 21 total; 71%). Cluster 34 contains the three genes for the p38 MAPK pathway, while Cluster 40 includes the three genes in each of the two ERK-class MAPK pathways (Additional file 1). These are ancient and conserved pathways with central roles in the cellular biology of many eukaryotic organisms, including baker’s yeast [31]. The co-clustering of mutants in the two ERK pathways was expected, as they have similar growth and morphological defects (Additional file 1). Overall, because genes lacking yeast orthologs predominate in our dataset, our analysis should reveal functions for genes found in *N. crassa* and other filamentous fungi that are not present in *S. cerevisiae*.

### Enzymes and transmembrane proteins in clusters

#### Enzymes

We used the list of genes compiled in previous work [32] to identify metabolic genes in the 40 clusters. Out of the 833 identified metabolic genes in the *N. crassa* genome, 88 were present in our dataset (11%; Additional fle 7). It should be noted that mutation of many metabolic genes results in auxotrophy in *N. crassa,* and since the knockout mutants were not cultured under conditions that would supplement all auxotrophs [11], those genes are not represented in our dataset. Therefore, it is likely that these 88 genes are either essential under other nutritional conditions not used in our experiments and/or are functionally redundant with another gene(s).

A total of six clusters (2, 8, 11, 14, 29 and 35) consisted of at least 15% metabolic genes, as compared to a global average of 6.9% (Additional files 1 and 7). All of these clusters except for Cluster 14 have reduced growth rates relative to wild type and, with the exception of Cluster 8, do not form ascospores (Table 2). These phenotypes may indicate diverse metabolic needs during hyphal growth and formation of the meiotic products, ascospores.

#### Predicted transmembrane proteins

**Fig. 5:**
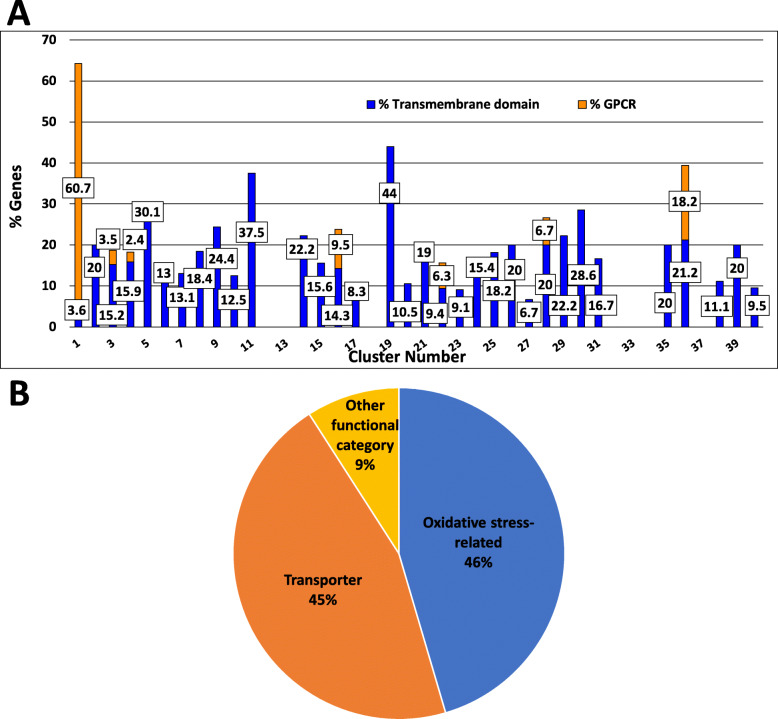
Genes encoding proteins with predicted transmembrane domain(s) and/or G protein coupled receptors (GPCRs). **a**. Transmembrane domains and GPCRs. The percentage of genes in each cluster encoding a protein with at least one predicted transmembrane domain is shown. The orange portion of each bar denotes the percent of genes in each cluster encoding transmembrane proteins that are also predicted G protein coupled receptors (GPCRs). Genes were retrieved using the “Protein targeting and localization - #TM Domains” tool at FungiDB. The numbers on the orange portion of the bars indicate the percent of proteins in a cluster that are GPCRs. The numbers on the blue portion of the bars indicates the percent of proteins in a cluster that have a predicted transmembrane domain. Raw data are available in Additional file 1. **b**. Functional categories of proteins with transmembrane domains in Cluster 19. Each “slice” of the pie represents the percentage of genes encoding proteins with transmembrane domains in the indicated functional category. Functional category data are available at https://elbe.hki-jena.de/fungifun/fungifun.php

We next identified proteins in the *N. crassa* genome and in clusters in our dataset that possess predicted transmembrane domains and/or are predicted G protein coupled receptors (GPCRs) (Fig. 5a). The outcome of the analysis showed that 1796 *N. crassa* genes (18.5% of the total) encode a protein with at least one transmembrane domain. Of the genes represented by the knockout mutants in this study, 228 (19.5%) encode proteins with predicted transmembrane domains. A total of 15 clusters have 20% or more genes encoding predicted proteins with transmembrane domains, with five clusters exceeding 30% (Clusters 1, 5, 11, 19 and 36; Fig. 5a). Within the transmembrane proteins, we also identified those that were predicted GPCRs [16]. Cluster 1 contained the largest percentage of GPCR mutants (17 genes of 28 total or nearly 61% of the cluster), followed by Cluster 36, with 18.2% (6 genes of 33 total) GPCR mutants (Fig. 5a).

There was no unifying group of phenotypes across the five clusters with the highest proportion of genes encoding transmembrane proteins (Table 2). However, 100% of the mutants in Clusters 19 and 36 possess tall aerial hyphae and 11 of 25 genes (44%) in Cluster 19 encode a protein with a predicted transmembrane domain (Fig. 5a). The 11 Cluster 19 genes encode either a transporter or an enzyme implicated in oxidative stress (Fig. 5b). ROS stress and a hyperoxidant state have previously been shown to be important signals for tissue differentiation at three different stages of aerial hyphae development [33, 34]. Oxidative stress releases repression by *conidial separation-1* (*csp-1*; a transcription factor), allowing ergosterol gene expression, a condition associated with aerial hyphae development [35]. The CSP-1 and CSP-2 transcription factors are both required for the formation of double-doublets during septation of conidiophores prior to production of the mature conidia [36]. However, *csp-2* (NCU06095), not *csp-1* (NCU02713), clusters with the mutants with tallest aerial hyphae, suggesting different molecular functions for these two transcription factors during asexual development. Other genes within the cluster may have regulatory roles in pathways involved with aerial hyphae development. One such gene is the predicted 5′ to 3′ exonuclease *exr-1* (NCU01643), which may negatively regulate some mRNAs that promote aerial hyphae development.

### Protein phosphorylation

#### Distribution of phosphorylated proteins

**Fig. 6:**
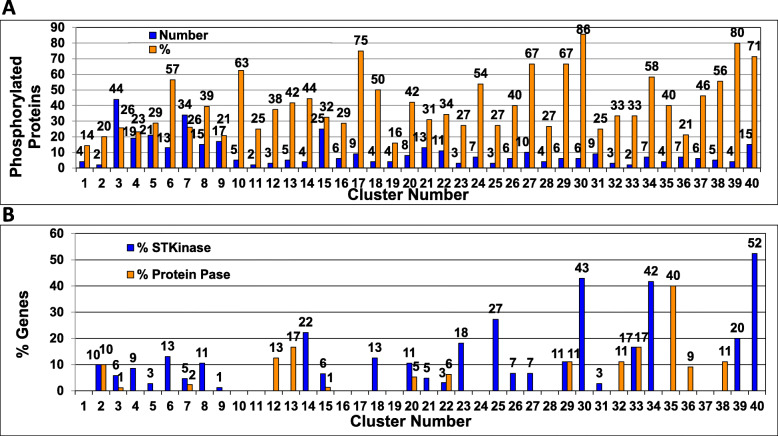
Protein phosphorylation-related genes. **a**. Assortment of phosphorylated proteins into clusters. Proteins that are phosphorylated during growth in submerged cultures with glucose as the carbon source were obtained from Ref. [37]. The number of phosphorylated proteins in each cluster (blue bars; actual number on top of bars), along with the % representation in each cluster (orange bars; % noted on top of bars) is shown. Data are taken from Additional file 1. The number above the blue bars represents the number of phosphorylated proteins in each cluster. The number above the orange bars indicates the percent of phosphorylated protein that make up each cluster. **b**. Distribution of serine-threonine protein kinases and serine-threonine or tyrosine protein phosphatases. The percentage of genes in each cluster encoding a serine-threonine protein kinase (STKinase) or serine-threonine or tyrosine protein kinase (Protein Pase) is shown. Data are taken from Additional file 1. The numbers above the blue bars indicates the percent of genes in each cluster that are serine/threonine protein kinases. The numbers above the orange bars represents the percent of genes in each cluster that are protein phosphatases

We used an available *N. crassa* phosphoproteome dataset [37] to ascertain whether the encoded proteins in the 40 clusters are phosphorylated when *N. crassa* is grown in liquid medium with glucose as the carbon source. Out of the 1168 genes in our study, 375 (32%) encode proteins that are phosphorylated, similar to the % in the entire genome (31%). Every cluster has at least two phosphorylated proteins and 14% or more of the proteins in each cluster are phosphoproteins (Fig. 6a). Clusters 10, 17, 27, 29, 30, 39 and 40 have 60% or more of their predicted proteins phosphorylated (Fig. 6a). Growth rate defects were observed for six of these clusters, five clusters have a conidiation defect and ascospores were either reduced or not formed in four clusters. Clusters 30 and 39 have more than 80% phosphorylated targets. These two clusters share reduced/severely reduced growth rates and reduced/severely reduced conidial abundance (Table 2).

#### Serine/threonine protein kinases and serine/threonine or tyrosine protein phosphatases

There are 86 genes encoding Serine/Threonine Protein Kinases (S/T Kinases) in *N. crassa*, with 77 mutants represented in our study [19]. Six clusters (14, 25, 30, 34, 39 and 40) have 20% or more S/T Kinase genes, with Clusters 30, 34 and 40 exceeding 40% (Fig. 6b). It is known that mutants lacking the mitogen-activated protein kinase (MAPK), MAPK kinase (MAPKK) and MAPKK kinase (MAPKKK) for the same signaling pathway possess similar phenotypes in *N. crassa* [38–42]. Thus, we expected to observe co-clustering of mutants for each of the three MAPK pathways. We noted that Cluster 34 contains the three kinases (*os-2*, NCU07024; *os-4*, NCU03071 and *os-5*, NCU00587) in the p38 MAPK osmosensing pathway (Additional file 1) [38, 39]. Similarly, five of the six kinases (*mak-1*, NCU09842; *mek-1*, NCU06419 *mak-2*, NCU02393 *mek-2*, NCU04612 and *nrc-1*, NCU06182) that comprise the two Extracellular Signal-Regulated Kinase (ERK) MAPK pathways in *N. crassa* are in Cluster 40 [40–42]. The MAPKKK for the cell integrity ERK pathway (*mik-1*, NCU02234) is in another cluster (Cluster 18), as the mutant has slightly taller aerial hyphae than the other two mutants in the same pathway (Additional file 1). Of note, Clusters 34 and 40 are among those with the highest proportion of kinase mutants (Fig. 6b), suggesting possible regulatory interactions between MAPKs and the other kinases in the cluster.

The *N. crassa* genome contains 30 genes encoding serine-threonine or tyrosine protein phosphatases, and 24 of these are available knockout mutants and were included in our analysis [18]. Protein phosphatases were not uniformly distributed throughout the clusters. There were numerous clusters (26 total) that lacked protein phosphatase genes (Fig. 6b). In contrast, 40% (4 genes of 10 total) of the genes in Cluster 35 were protein phosphatases, and this cluster had reduced growth rate and reduced numbers of protoperithecia and perithecia.

### Identification of cluster genes regulated by MAPKs in *N. crassa* and related fungi

As mentioned above, we noted that with one exception, all three genes for each of the three MAPK cascades clustered together. Considering the importance of these three evolutionarily conserved MAPK pathways in fungi [43], we mined for potential targets in the clusters using a variety of publicly available datasets.

**Fig. 7:**
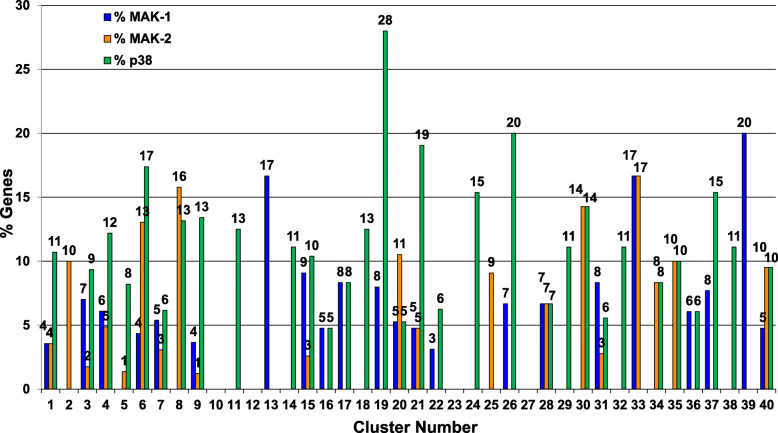
Cluster genes dependent on the three MAPKs: MAK-1, MAK-2 and OS-2. Data for identification of genes with down-regulated expression in a *N. crassa* Δ*mak-1* mutant were obtained from Ref. [44]. For mining cluster genes encoding transcriptional or phosphorylation targets of MAK-2 in *N. crassa,* targets were compiled from four datasets, including microarray analysis of genes down-regulated in a Δ*mak-2* mutant [41] or down-regulated in a strain expressing the *mak-2*^Q100G^ strain in the presence of inhibitor [45], and MAK-2 phosphorylation targets identified during phosphoproteomics studies with the *mak-2*^Q100G^ strain with inhibitor [46, 47]. Cluster genes encoding *N. crassa* orthologs of transcriptional targets of the p38 MAPK (*os-2*) in *Cryptococcus neoformans* and *Aspergillus fumigatus* were obtained using data from Ref. [48, 49]. *N. crassa* orthologs in the combined dataset were retrieved using the “Transform by Orthology” tool at FungiDB. The numbers above the blue, orange and green bars indicate the percent of genes in each cluster that are either regulated by MAK-1, MAK-2 and the p38 MAPK (OS-2), respectively. Raw data are available in Additional file 7

In order to identify genes regulated by the MAK-1 ERK MAPK, we first analyzed results from microarray analysis of the Δ*mak-1* mutant in *N. crassa* [44]. Of the 424 genes down-regulated in the Δ*mak-1* mutant, 57 were included in our study (Fig. 7). Cluster 40, which contains the three MAK-1 MAPK pathway kinases, has a relatively low percentage of genes that are targets (< 5%; Fig. 7). In contrast, there were three clusters with > 15% MAK-1 target genes: Clusters 13 (2 genes of 12 total), 33 (1 gene of 6 total) and 39 (1 gene of 5 total). These three clusters share reduced/severely reduced growth rates and varying degrees of sexual cycle defects (Table 2). These phenotypes are less severe examples of the phenotypes possessed by Δ*mak-1* mutants.

To determine putative targets of the ERK MAPK, MAK-2, we took advantage of two transcriptomic datasets, one for a Δ*mak-2* mutant [41] and the second for a strain expressing a *mak-2* inhibitable allele (*mak-2*^Q100G^) grown in the presence of inhibitor [45]. We also queried two phosphoproteomics datasets, both obtained after treatment of the *mak-2*^Q100G^ strain with inhibitor [46, 47]. There were 39 genes in common between our phenotypic dataset and these four combined datasets (Fig. 7). Clusters 8 and 3 contained more than 15% of the target genes. The only phenotype in common for these two clusters is reduced growth rate (Table 2). Cluster 40, containing the three kinases of the MAK-2 pathway, had 10% of its members as predicted targets.

There is currently no publicly available transcriptomic data for a Δ*os-2* or other p38 MAPK pathway mutant in *N. crassa*. Therefore, we turned to gene expression studies of the orthologous Δ*os-2* mutant in two filamentous fungi: microarray analysis of a Δ*hog1* mutant in *Cryptococcus neoformans* [48] and an RNA-seq experiment using an *Aspergillus fumigatus* Δ*sakA* mutant [49]. Of the 401 genes regulated by *hog1* in *C. neoformans*, 296 had orthologs in *N. crassa* and 37 were represented in our phenotype dataset **(**Fig. 7**)**. The *A. fumigatus* RNA-seq analysis produced more hits, with 654 genes regulated by *sakA*, 574 of which have an ortholog in *N. crassa* and 96 that were included in our study **(**Fig. 7**)**. Analysis of these two combined datasets revealed that Cluster 19 had the highest percentage of p38 MAPK targets (7 genes of 25 total; 28%). This cluster also has the tallest aerial hyphae of any cluster. Other clusters with a relatively high proportion of target genes (> 15%) were 6 (4 genes of 23 total), 21 (8 genes of 42 total), 26 (3 genes of 15 total) and 37 (2 genes of 13 total). These clusters all have reduced or severely reduced growth rates and reduced aerial hyphae height.

Osmotic Sensitive (OS) pathway mutants have reduced growth rates, but normal aerial hyphae height and do not produce female sexual structures. The lack of concentration of possible OS-2 pathway targets in Cluster 34 (containing the three OS-2 pathway kinases) supports a mechanism in which the diverse functions of the p38 MAPK pathway are carried out by different groups of genes. The observation of slower growth rate in mutants lacking p38 MAPK target genes is consistent *N. crassa os-2* pathway mutant defects (Table 2; Additional file 1). It is of interest that of the clusters with a significant percentage of targets, only Cluster 37 has a sexual cycle phenotype, reduced ascospore production.

We also interrogated our dataset to identify genes that are targets of multiple MAPK pathways (Additional file 8). A total of 188 genes encoded targets of at least one MAPK pathway. Of these, 18 are targets of two different MAPK pathways and one is a target of three pathways (Fig. 7). The triple target is in Cluster 3, NCU03753, *clock-controlled gene-1* (*ccg-1* [50, 51];. Cluster 3, which has wild type characteristics, also has the largest number of single MAPK targets (26 genes; Fig. 7). The clusters with the greatest number of genes that are targets of two different MAPK pathways are 7 and 15 with three targets each (Fig. 7). These last two clusters have similar phenotypes, with reduced growth rates (Table 2).

### Transcriptional regulation

#### Distribution of transcription factors across phenotype clusters

*N. crassa* has 314 genes encoding transcription factors [17], with 242 (77% of the total) represented in our dataset. Transcription factors represent ~ 20.7% of the genes in our dataset and the average percentage of genes in a cluster that are transcription factors is 19.9%. Cluster 10 had the highest percentage of transcription factors, with 75% (Additional file 7) and mutants in this cluster have a severe reduction in hyphal growth rate (Table 2). Interestingly, none of the Cluster 10 genes are targets of the MAK-1, MAK-2 or OS-2 MAPKs (Fig. 7).

#### Co-expression of genes in clusters

We utilized publicly available RNA-seq and microarray data sets for wild-type *N. crassa* to determine whether genes in the same phenotypic cluster are co-expressed. The two RNA-seq datasets are time courses during sexual development [52] or conidial germination [53], while the two microarray datasets are time courses during conidiation [54] or colony development [55]. Expression data is available for ~ 99% of the cluster genes in the two RNA-seq datasets, but only 46.3% [54] and 49.7% [55] of the cluster genes were represented in the two microarray datasets. We did not analyze phenotypic clusters in which less than three genes had expression data.

Initial visual inspection of expression trends in each cluster using line plots showed few examples of co-expression (Additional file 9). However, in clusters with more than five genes, it becomes difficult to visually determine expression patterns. Therefore, we attempted to fit linear, polynomial and sinusoidal models to the expression data for each phenotypic cluster. However, no model fit the data beyond an r-squared value of 0.2 (Additional file 10), supporting more than one expression pattern per cluster.

We next turned to K-means clustering to separate genes into discrete expression profiles for each mRNA time course and then compared the genes in these profiles to those in the 40 phenotypic clusters (See Methods). Using Within sum of squares [56], Gap statistic [57] and Davies-Bouldin index [58] as measures of cluster quality, we determined that 6–8 expression profiles provided sufficient quality clusters, depending on the dataset. We then determined the number of expression profiles that were present in each phenotypic cluster. We focused on phenotypic clusters in which one expression profile could be assigned to 40% or more of the genes in that cluster.

**Fig. 8:**
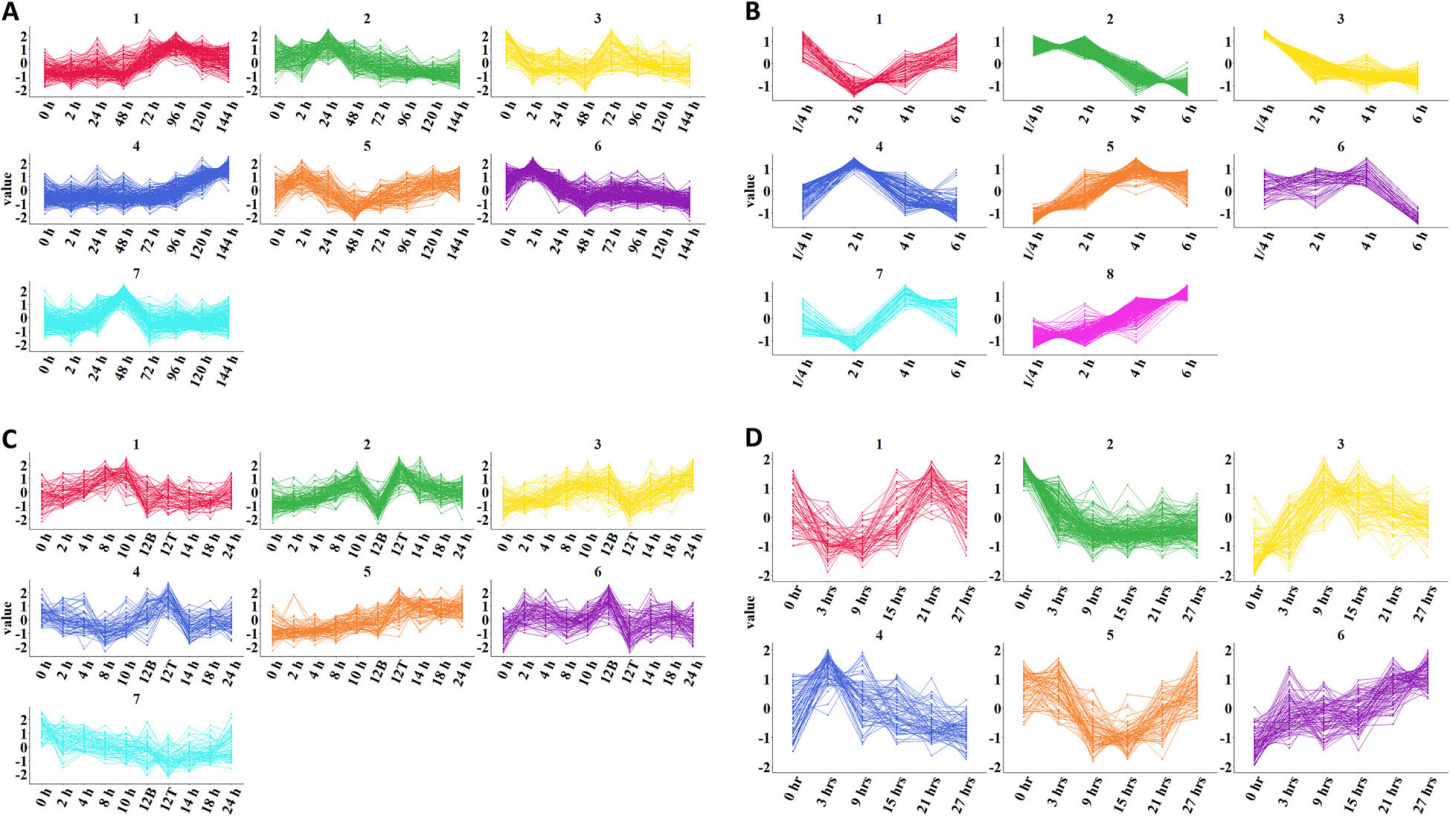
K-means clustering of transcriptomic data from wild type for genes in the phenotype dataset. The expression values for each gene in the phenotype dataset were standardized to have a mean of 0 and a standard deviation of 2. Genes were partitioned into the indicated number of expression profiles using K-means clustering. **a**. RNA-seq data from a sexual cycle time course. RNA-seq data for eight time points were obtained from Ref. [52]. Genes were partitioned into seven expression profiles. **b**. RNA-seq data from a time course of conidial germination. RNA-seq data for four time points were obtained from Ref. [53]. Genes were partitioned into eight expression clusters. **c**. Microarray data from a time course of conidiation. Microarray data for 10 time points were obtained from Ref. [54]. Genes were partitioned into seven expression clusters. **d**. Microarray data during a colony development time course. Microarray data for six time points were obtained from Ref. [55]. Genes were partitioned into six expression clusters

The genes for the sexual development [52], conidial germination [53], conidiation [54] and colony development time courses [55] were divided into seven, eight, seven and six different expression profiles, respectively (Fig. 8a-d). The average number of expression profiles per cluster ranged from 4.1 to 6.1, revealing high diversity in expression profiles per cluster (Additional file 9). When we focused on those clusters with a phenotype related to their respective datasets, the average number of profiles per cluster only decreased slightly, to a range of 3.9 to 5.9. In addition, there were no expression profiles that were exclusively correlated with a specific phenotype.

While the average number of expression profiles per cluster was high, there were clusters where more than 40% of genes shared the same expression profile. We investigated whether any of these dominant expression profiles were present in more than 50% of the phenotypic clusters. For sexual development and conidiation, no one expression profile was represented in more than 50% of clusters with a dominant expression profile (Additional file 9). However, for conidial germination and colony development there were expression profiles representing 77 and 86%, respectively, of the dominant expression profiles in the clusters (Additional file 9). The two dominant expression profiles were expression profile 3 during conidial germination (peak expression early) and expression profile 6 during colony development (peak expression at the hyphal tip) (Fig. 8a,c). Whereas there was little correlation between any expression profile and phenotype for sexual development and conidiation, there was some correlation between defects in growth rate and elevated expression at the hyphal tip or early during conidial germination, as those phenotypes and expression profiles most often grouped together.

#### Cluster genes co-expressed across multiple datasets

Interestingly, three phenotypic clusters (14, 30 and 33) contain several genes that are consistently co-regulated across multiple datasets. In Cluster 14, the transcription factors *bek-1* (NCU00097) [59] and *bek-2* (NCU07139), the guanosine diphosphatase *gda-1* (NCU03713), hypothetical protein NCU06390 and the S/T kinase *stk-53* (NCU09064) share the same expression profile during conidial germination and sexual development (Additional file 11). In Cluster 30, the transcription factors *vel* (NCU00406) and *ada-19* (NCU04459) are co-expressed during conidiation and conidial germination (Additional file 11). In Cluster 33, four genes are co-expressed during sexual development and conidial germination: two signaling genes (the phosphatase *tng* (NCU0436) and kinase *div-4* (NCU04426), a chromatin remodeling factor *crf4–1* (NCU03875) and a gene implicated in mRNA stability (NCU07874). With regards to MAPK signaling, the p38 MAPKK *os-5* and MAPKKK *os-4* are co-expressed during colony development and conidiation (Additional file 11). *mak-1* and *mak-2* are co-expressed in every time course (Additional file 11) and *os-2* is co-expressed with *mak-1* and *mak-2* during colony development (Additional file 11).

Taken together, the results from our analysis of transcriptional data indicate that in general, no single expression profile correlates with a given phenotype. These results support earlier observations that elevated expression of a gene during a developmental time course does not necessarily correlate with an observable phenotype for the corresponding mutant in *N. crassa* [60, 61].

### Pathway prediction utilizing yeast ortholog interaction data

**Fig. 9:**
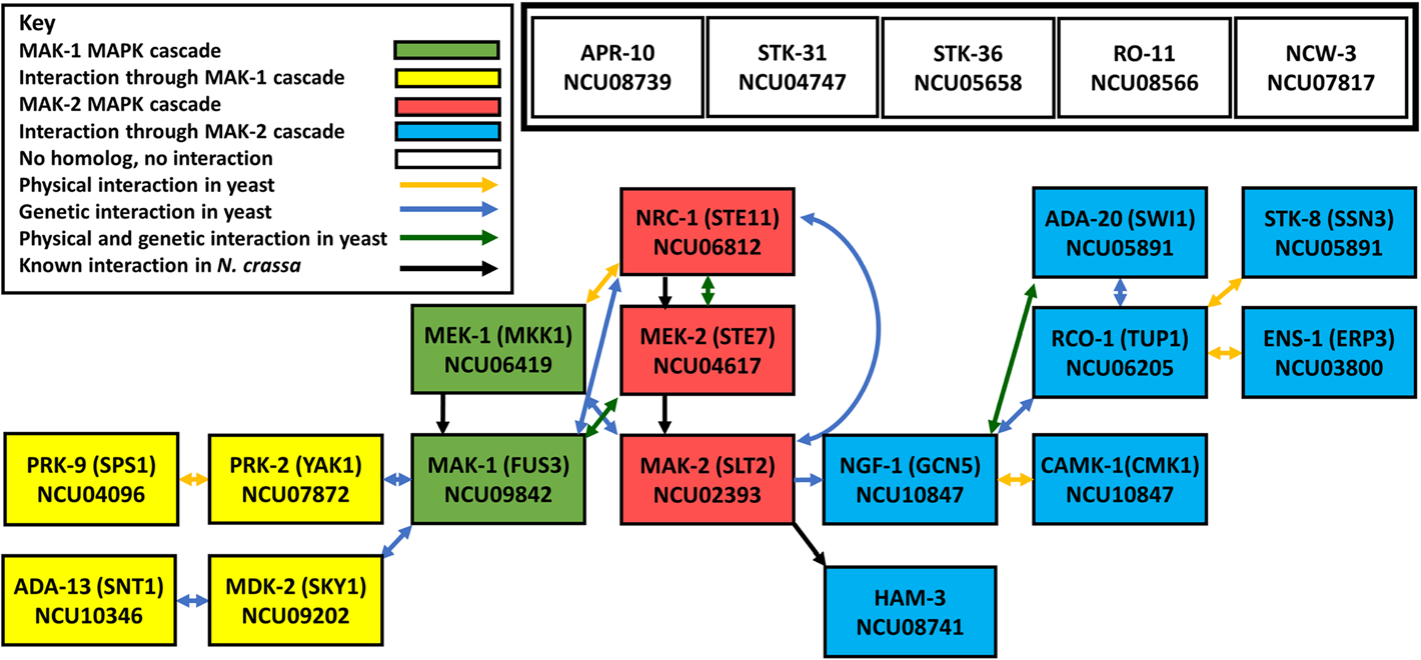
Model showing predicted pathway(s) for genes that co-cluster with the ERK MAPK genes in Cluster 40. The MAK-1 and MAK-2 ERK MAPK cascade genes are represented by green and red shading, respectively. *S. cerevisiae* interaction data for genes in Cluster 40 were obtained from www.yeastgenome.org. Physical interactions, genetic interactions, or physical and genetic interactions between the indicated genes/gene products in yeast are shown by yellow, blue, or green lines, respectively. Genes with known interactions in *N. crassa* are connected by black lines. Genes that interact with the MAK-1 cascade kinases are shaded in yellow, while those that interact with components of the MAK-2 pathway are indicated with blue shading. Genes with no ortholog in yeast and no known interaction in *N. crassa* are represented by the white rectangles

In order to determine possible targets and interactors in the three MAPK pathways, we identified genetic and physical interactions between all yeast orthologs of genes in the two clusters. Of the genes that co-clustered with the two ERK MAPK pathways, 11 of 16 had a yeast ortholog. All but one yeast ortholog has evidence of either genetic and/or physical interactions with the other genes in the cluster. Based on known genetic and physical interactions in yeast, we predict that there may be two pathways associated with the two ERK MAPK signaling cascades (Fig. 9). One possible pathway runs from MAK-2 to NGF-1 through a negative genetic interaction. NGF-1 then has various genetic and physical interactions with CAMK-1, RCO-1 and ADA-20 (Fig. 9). The other pathway flows from MAK-1 to PRK-2 and MDK-2 through negative genetic interactions (Fig. 9). There are five cluster genes that do not have a yeast ortholog, including two serine/threonine kinases (*stk-31* and *stk-36*), an endothiapepsin (*apr-10*), a non-anchored cell wall protein (*ncw-3*) and a protein with a tropomyosin domain (*ro-11*). These genes may represent additional pathways and/or interactors downstream of the ERK MAPKs that are not found in *S. cerevisiae*.

**Fig. 10:**
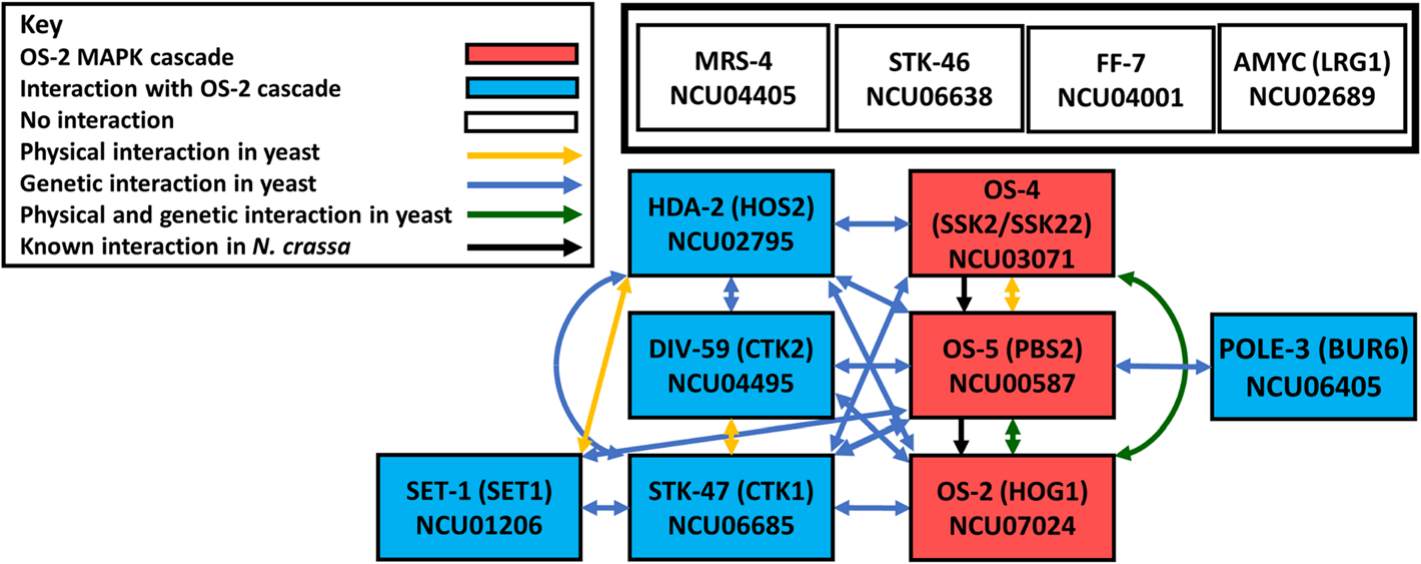
Model for predicted pathway(s) including genes that co-cluster with the p38 MAPK genes in Cluster 34. The OS-2 MAPK cascade genes are represented by red rectangles. *S. cerevisiae* interaction data for genes in Cluster 34 were obtained from www.yeastgenome.org. Physical interactions, genetic interactions, or physical and genetic interactions between the indicated genes/gene products in yeast are indicated by yellow, blue, or green lines, respectively. Genes with known interactions in *N. crassa* are connected by black lines. Genes that interact with the OS-2 cascade kinases are represented by blue rectangles. Genes with no known interaction in *N. crassa* or *S. cerevisiae* are represented by the white rectangles

Nine of the 12 genes in Cluster 34, which contains the genes in the p38 MAPK cascade, have a yeast ortholog. There is substantial evidence for interaction between the yeast orthologs in this cluster. The data also suggests a large amount of crosstalk between the MAPK pathway and three genes that co-clustered (Fig. 10); orthologs of *hda-2*, *div-59*, and *stk-47* each exhibit a genetic relationship with at least two out of three of the genes in the p38 MAPK cascade (Fig. 10). Interestingly, there was one gene with a yeast homolog (*amyc*) that does not interact with any orthologs of Cluster 24 genes in yeast. There are three genes with no yeast ortholog in the cluster; a serine/threonine kinase (*stk-46*), a transcription factor (*ff-7*) and a mago nashi protein (*mrs-4*). These last four genes may be unique targets of the p38 MAPK pathway in *N. crassa*.

## Discussion

In this study, we took advantage of a large dataset of phenotypic data for 10 traits obtained from 1168 knock out mutants in *N. crassa*. The distribution of genes across linkage groups and functional categories was representative of the entire genome. We found that the majority of genes lack an orthologous gene in yeast and, therefore, many relationships and genes explored in this study cannot be studied in *S. cerevisiae*. With this broadly representative dataset, we tested several algorithms and two distance measures to determine the optimal way to cluster our phenotypic data. We developed a novel approach to measure cluster quality, using relative standard deviation for each quantitative trait and the average percent consensus (the average percent of the most prevalent category per categorical trait), in order to determine biological relevance of the clusters.

Previous studies have focused on using a single hierarchical clustering method for analyzing phenotypic data. For example, in *S. cerevisiae*, data was collected for 4281 gene deletion mutants subjected to 51 different environmental stressors [20]. All phenotypes were quantitative and were clustered using two-way unsupervised, un-centered hierarchical agglomerative clustering, with a distance matrix generated using Pearson’s correlation [20]. In *Fusarium graminearum,* 17 different phenotypes were collected for 657 transcription factor knockout mutants [21]. Continuous and categorical phenotypes were converted to a number scale and correlations between phenotypes were calculated using Pearson’s correlation coefficient. In *Cryptococcus neoformans*, a study analyzed 30 different phenotypes for 129 kinase mutants [22]. Categorical and continuous phenotypes were converted to a numeric value. A distance matrix was generated with Pearson’s correlation minus one and a hierarchical agglomerative clustering algorithm was applied to generate clusters [22].

Our initial attempts to cluster relied on conversion of ordinal traits to a numerical scale. However, only four of the eight categorical phenotypes in our dataset were amenable to this conversion. Using the converted dataset, both a hierarchical method (Hierarchical Agglomerative Clustering [22];) and a partitional approach (K-means) [25] were tested for their ability to cluster genes, with unsatisfactory results in terms of large standard deviations. Therefore, we turned to distance matrices and algorithms that can utilize categorical data. Of these, the algorithm that performed the best according to our criteria was Partitioning Around Medoids (PAM). For optimal clustering, we weighted the data in the distance matrix before applying PAM. To our knowledge, PAM has not previously been used to cluster phenotypic data in fungi.

We identified two clusters in which more than 40% of the genes in the cluster encode proteins with predicted transmembrane domains. One of the clusters, Cluster 1, is comprised mostly of G-protein coupled receptors, which have been previously shown to regulate aerial hyphae height [16]. In the other cluster (Cluster 19), a majority of genes are involved in oxidative stress regulation or ion transport. ROS stress and a hyperoxidant state are important signals during aerial hyphae development [33, 34]. Additionally, turgor pressure has been shown to be vital for hyphal extension [62]. Indeed, the same forces must be applied to grow vertically as well, with a combination of turgor pressure and secretion of hydrophobins (*eas*, NCU08457) [63]. Thus, Cluster 19 genes highlight an interesting correlation between membrane-associated proteins and aerial hyphae height in *N. crassa.*

We investigated whether gene expression was correlated with some or all of the phenotypes we observed. There was some correlation between expression at hyphal tips and defects in hyphal growth rate. However, results using most of the gene expression datasets did not reveal a strong correlation between an expression profile and a phenotype. The lack of correlation is not particularly surprising, as it has been observed that mutation of several genes that are highly expressed during conidiation did not lead to a phenotype during conidiation [60, 61]. However, the lack of correlation could also be explained by differences in experimental conditions between our phenotypic analysis and those used to isolate RNA for transcriptional profiling. There are a limited number of time-course expression datasets that are publicly available for *N. crassa,* and there was no dataset with conditions or developmental processes that perfectly matched those in our study.

Mitogen-activated protein kinase (MAPK) pathways are conserved in eukaryotes, from unicellular organisms such as yeast, to metazoans, including humans [64, 65]. In animals, MAPKs regulate gene expression, mitosis, movement, metabolism, and programmed cell death [64]. In plants, MAPK cascades control cell division, growth and development, and regulate resistance to pathogens, insect herbivores, and abiotic stress [66]. In fungi, including saprobes and pathogens, MAPKs have been found to be involved in control of morphogenesis, development, virulence, cell wall biogenesis and stress responses [43, 67]. In *N. crassa*, mutation of any of the three genes for each of the three MAPK cascades leads to severe defects in growth rate, asexual development, sexual development and failure to form conidial anastomosis tubes [19, 68]. In addition, the MAK-1 and MAK-2 pathways are both involved in cell wall integrity, with the MAK-1 pathway more important for this process [42]. Mutants lacking any of the three genes in the OS-2 MAPK pathway have near-normal growth rates, but possess defects in conidiation and sexual development [19].

Analysis of transcriptional and proteomic targets of the three MAPK signaling pathways revealed that targets are distributed throughout the clusters, supporting the idea that the functions of these pathways are carried out by different groups of genes. We also determined that several MAPK targets are actually genes/proteins in a different MAPK cascade. This is not surprising, as MAPK pathways are not necessarily linear and independent, and there is evidence showing significant amounts of crosstalk in mammalian cell lines [69, 70]. In *S. cerevisiae*, crosstalk has been shown between the osmosensing (HOG1) and the cell wall integrity MAPK pathways during heat stress and mating in specific mutant backgrounds [71, 72]. Crosstalk has also been reported in *Aspergillus fumigatus* between the cell wall integrity and spore development pathways [73]. Interestingly, we found that across all four RNA expression datasets, two of the three terminal MAPKs in *N. crassa* (*mak-1* and *mak-2*) were always co-expressed, implying some amount of co-regulation. To our knowledge, no other study has reported transcriptional co-expression of these genes.

We took advantage of the extensive interaction data available for *S. cerevisiae* to build predicted pathways for the genes that co-clustered with the ERK and p38 MAPKs. We discovered that all genes with a yeast homolog in the cluster containing the two ERK MAPK cascades showed an interaction with at least one other gene in the cluster. For the genes that co-clustered with the p38 MAPK cascade, all but one interacted with at least one other gene in the cluster. Interestingly, there was linear flow through the ERK MAPK signaling cascades, with the MAPKKK and MAPKKs only interacting with the MAPKK or MAPK from the same or the second pathway. The rest of the interacting genes formed a network that emanated from one of the terminal MAPKS (MAK-1 or MAK-2) (Fig. 9). In contrast to the interactions observed in the ERK MAPK cluster, there were three genes (*hda-2*, *div-59* and *stk-47*) that interacted with at least two of the three core kinase genes in the p38 MAPK cascade and also exhibited many interactions with each other (Fig. 10). Further work is needed to confirm these models, including probing physical interactions through the yeast two-hybrid assay or pull-down approaches, as well as tests of genetic epistasis.

## Conclusions

A major goal of this study was to identify a clustering method that can utilize the large amount of phenotypic data that is available for filamentous fungi. We demonstrate that PAM clustering can be used to generate new hypotheses about cellular pathways in *N. crassa*, as highlighted by our models for the ERK and p38 MAPK pathways. We believe that data for additional mutants can be incorporated in the future and the resulting dataset re-clustered to reveal additional relationships between genes. Furthermore, this methodology can be extended to other phenotypes, such as those obtained from chemical and nutritional screens. For example, we have previously reported phenotypes for mutants lacking *N. crassa* phosphatases [18], S/T Kinases [19] and GPCRs [16] after exposure to hyperosmotic (NaCl and sorbitol) and oxidative (menadione and peroxide) stress, to agents that perturb the cytoskeleton or other cellular functions, and after growth on different media. Once data for a large number of mutants is available, the appropriate traits can be included in the clustering analysis. These and other approaches can be used in the future to leverage this important genetic resource for *N. crassa* and other filamentous fungi.

## Methods

### Data sources and curation

Knockout mutants were produced during the Neurospora Genome Project (https://geiselmed.dartmouth.edu/dunlaploros/genome/) in the Dunlap or Borkovich laboratories [12] and deposited at the Fungal Genetics Stock Center [13]. Most phenotypic data for the mutants were obtained by undergraduate students in summer research programs or during courses at the University of California, Los Angeles, the University of California, Riverside (UCR), Texas A&M University and the University of Manchester, UK. Phenotypic data for kinase, phosphatase, GPCR and transcription factor mutants have been previously published [16–19]. Data were initially deposited at the Broad Institute-MIT (https://www.broadinstitute.org). After downloading and curation, the data were then migrated to FungiDB (fungidb.org) [74, 75]. All mutants with complete data for the 10 chosen traits (see below) were included in our analysis.

The methods used for phenotypic analysis were as previously described [11, 15–19] and will be briefly summarized here (also see “Metadata” tab in Additional file 1). Similar to recent publications, we have omitted measurements of pigmentation and aerial hyphae height on yeast extract-containing medium from our analysis [16, 17]. Knockout mutants were either obtained from the Fungal Genetics Stock Center (FGSC; Kansas State University, Manhattan, KS; http://www.fgsc.net) or produced in the Borkovich laboratory using methods described in [11]. Near-isogenic wild-type strains FGSC4200 and/or FGSC2489 (obtained from the FGSC) were used as controls.

#### Hyphal growth rate

The apical extension rate of basal hyphae was measured using glass or disposable race tubes [76, 77] containing Vogel’s minimal agar medium (VM [78];. Tubes were inoculated at one end and were incubated at 25 °C under ambient light conditions [15] or in the dark [11, 18, 19]. Before marking the growth front, tubes were grown overnight to eliminate effects on growth rate due to the age of the culture or germination defects. The total growth in mm was measured for each time point and a plot of mm vs. time used to determine growth rate. A minimum of four replicates with growth rates with R squared values greater than 0.95 were used to obtain the average growth rate for each strain. Binned data from [11] and/or the Broad Database were averaged to allow comparison to actual growth rate measurements obtained for some mutants. The wild-type growth rate range was 75–85 mm/day (Metadata tab in Additional file 1).

#### Asexual development

The height of aerial hyphae was measured in liquid standing cultures containing VM. Tubes were incubated statically (typically in the dark) at 25 °C for 3–4 days. Total height (in mm) was recorded. A minimum of four replicates was analyzed for each strain. The average value in mm was reported. As for growth rate, binned data were averaged. The wild-type range was 30–45 mm (Metadata tab in Additional file 1). For semi-quantitative analysis of conidia number and morphology, slant tubes (13x100mm) containing 3 ml of VM agar medium were inoculated with strains and grown at room temperature for 6 to 8 days under ambient light conditions [15] or for 3 days in the dark at 30 °C and 4 days in the light at room temperature [11, 18, 19]. Production of conidia was scored visually, for amount and morphology. A minimum of four replicates was analyzed for each strain. See metadata tab in Additional file 1 for the scoring rubric for conidia number and morphology.

#### Sexual development

Synthetic crossing medium (SCM) [78] agar slant tubes were inoculated with the various strains. After 7–8 days of incubation at room temperature in constant light, cultures were scored for the number and morphology of protoperithecia by inspection using a stereomicroscope. At least four replicates were scored for each strain. The 7–8 day old SCM cultures from the protoperithecial scoring were fertilized using a suspension of wild-type conidia of the opposite mating type. Cultures were returned to the same conditions used for protoperithecial development. After seven more days (~ 2 weeks total), cultures were scored for the number and morphology of perithecia using a stereomicroscope. The 2-week-old SCM cultures from the perithecial scoring were returned to the same culture conditions. After seven more days of incubation (~ 3 weeks total), cultures were scored for the number and morphology of ascospores using a stereomicroscope. Perithecial beak morphology was also scored at this point. See metadata tab in Additional file 1 for scoring rubric for protoperithecia, perithecia and ascospore number and morphology.

### Clustering approaches

To uncover new biological pathways, we grouped mutants with similar phenotypes into clusters using several algorithms for hierarchical and partitioning clustering: Pearson’s Correlation Coefficient [23], K-means [25], Factor Analysis of Mixed Data (FAMD) [26], Ward’s minimum variance (Ward’s) [27] and Partitioning Around Medoids (PAM) [24]. Initial clustering was performed with algorithms and distance matrices that cannot utilize non-numeric data. Data was initially transformed with the semi-quantitative categorical variables (ordinal categories) being converted to a value between 0 and 1.5 based on severity of the phenotype. The scale was chosen based on increments of 0.25 with 6 categories, from not formed (0), severely reduced (0.25), reduced (0.5), slightly reduced (0.75), normal (1.0), and increased (1.5). These values are based on the approximate quantitation applied during the scoring (see Metadata in Additional file 1). A one minus Pearson’s Correlation Coefficient distance matrix [23] was created using the Factoextra package in R [79]. The Pearson’s distance matrix was used as the input for Hierarchical Agglomerative Clustering (HAC) with complete linkage [22]. K-means clustering [25] was performed using base R (https://www.r-project.org/) with the converted dataset as the input.

Further clustering was performed with algorithms and distance matrices that can handle mixed categorical and numeric data. Factorial Analysis of Mixed Data (FAMD) [26] was performed using the FactoMineR package in R [80]. The non-converted dataset with categorical data left as-is (including both ordinal and categorical data) was used as the input. A Gower’s distance matrix metric [30] was created using the clustering package in R [29]. The Gower’s matrix was used as input for Ward’s minimum variance clustering algorithm [27] and Partitioning Around Medoids (PAM) clustering algorithm [24]. Both algorithms were run in R using the clustering package [29]. All packages and information needed to run this algorithm are available or easily added to R (https://r-project.com) and analysis scripts used in this study are available in Additional file 2.

In order to judge the biological relevance of the clusters, we determined the average relative standard deviation for the two continuous traits (basal hyphae growth rate and aerial hyphae height) and the average percent consensus for the categorical traits (all other phenotypes). To determine the relative standard deviation for the two continuous phenotypes we first calculated the standard deviation for each cluster and then divided that standard deviation by the cluster mean to determine the relative standard deviation. In order to create a composite relative standard deviation for the “run” (e.g., 21 total clusters and 22 total clusters are two different “runs”), we calculated the average relative standard deviation for all clusters in that run. For categorical phenotypes, we first calculated the percentage of each category/phenotype for the cluster and then identified the most prevalent category (category with the highest percent representation). For example, if a cluster contained 10 genes/mutants, with six having reduced conidial abundance, then that cluster would have a value of 60% for conidial abundance. This was repeated for all clusters in the run. We then determined an average percent consensus by calculating the average representation (%) of the most prevalent category for all clusters in the run. The relative standard deviations and the average percent consensus were then averaged across the two continuous phenotypes and the eight categorical phenotypes to arrive at two composite values for each run utilizing each clustering approach.

### Analysis of specific classes of cluster genes

Functional catalogue (Funcat) analysis was performed on *N. crassa* genes using FungiFun (https://sbi.hki-jena.de/fungifun/) to test for enrichment of specific functions among the gene clusters. A list of 5781 genes that are annotated with functional categories was obtained from the FungiFun website. Duplicates were removed, yielding a final list of 5082 genes. All genes not in the list were categorized as “Unclassified”. The *p*-value significance level was set to 1 to capture all possible associations of genes with functional categories. The annotation type was set to “Use also indirectly annotated top categories” to simplify the functional categories to their top-level category.

*N. crassa* genes with orthologs in other fungi were identified using the “Transform by Orthology” tool at FungiDB (FungiDB.org). Genetic and physical interactions between orthologs and other genes/gene products in baker’s yeast were identified using the “interactions” tab at the Saccharomyces Genome Database (yeastgenome.org) [81]. *N. crassa* metabolic genes were obtained from [32]. Genes encoding proteins with secretion signals and/or transmembrane domains were retrieved using the “Protein targeting and localization” tool at FungiDB. Predicted proteins that are phosphorylated in *N. crassa* were obtained from [37].

Targets of the two Extracellular-signal Regulated Kinase (ERK) class Mitogen-Activated Protein Kinase (MAPK) cascades were identified in publicly available phosphoproteomic or transcriptomic datasets for *N. crassa* (whenever possible) or other fungi. For the MAK-1 MAPK pathway, clusters were checked for misregulated genes using microarray data for a Δ*mak-1* knockout mutant [44]. For the MAK-2 MAPK cascade, microarray data from a Δ*mak-2* mutant [41] or a strain expressing a *mak-2* inhibitable allele (*mak-2*^Q100G^) in the presence of inhibitor [45], as well as phosphoproteomics data from two additional studies using the *mak-2*^Q100G^ strain in the presence of inhibitor [46, 47] were utilized. Because there currently is no transcript profiling data available for mutants lacking genes in the p38 MAPK OS-2 pathway in *N. crassa*, RNA-seq or microarray datasets were analyzed for genes controlled by the *os-2* orthologs *sakA* or *hog1* in *Aspergillus fumigatus* or *Cryptococcus neoformans*, respectively [48, 49].

Publicly available transcriptomics datasets for wild-type *N. crassa* were used to compare gene expression trends to phenotypes in clusters. These included two microarray datasets, corresponding to time courses of macroconidiation [54] and colony growth [55], as well as two RNA-seq datasets for time courses during the sexual cycle [52] and conidial germination [53]. Expression data was scaled to values between − 2 and 2 to give comparable relative expression per gene. K-means clustering [25] was then performed to produce expression profiles. Comparisons between the phenotypic clusters and the expression profiles were made to check for relationships between mRNA expression and phenotype.

## Supplementary information

**Additional file 1.**

**Additional file 2.**

**Additional file 3.**

**Additional file 4.**

**Additional file 5.**

**Additional file 6.**

**Additional file 7.**

**Additional file 8.**

**Additional file 9.**

**Additional file 10.**

**Additional file 11.**

## Data Availability

All data used for this study (including phenotypes for individual mutants) are available in the Additional files, the main tables and figures of the manuscript, or at the links in the table, below. Phenotypes for *N. crassa* mutants are also available on the gene pages on FungiDB.org. *N. crassa* strains are available from the Fungal Genetics Stock Center (fgsc.net) or upon request. **Table:** 

**Phenotyping dataset**	https://fungidb.org/fungidb/app/record/dataset/DS_c334877c42
**Functional Categories**	https://elbe.hki-jena.de/fungifun/annotation_info.php Search Neurospora crassa
**Metabolic gene dataset**	https://journals.plos.org/ploscompbiol/article/file?id=10.1371/journal.pcbi.1003126.s001&type=supplementary
**Phosphorylated proteins dataset**	http://proteomecentral.proteomexchange.org/cgi/GetDataset?ID=PXD013964
**Sexual development RNAseq time course**	https://www.ncbi.nlm.nih.gov/geo/query/acc.cgi?acc=GSE41484
**Conidial germination RNAseq time course**	https://www.ncbi.nlm.nih.gov/geo/query/acc.cgi?acc=GSE101412
**Conidation microarray time course**	http://bioinfo.townsend.yale.edu/browse_detail.jsp?exptid=64
**Colony growth microarray time course**	https://ec.asm.org/content/7/9/1549/figures-only#fig-data-additional-files
**Microarray analysis of the Δ*****mak-1*****mutant**	https://www.ncbi.nlm.nih.gov/geo/query/acc.cgi?acc=GSE41778
**Microarray analysis of the Δ*****mak-2*****mutant**	https://www.genetics.org/content/170/3/1091
**Microarray analysis of the*****mak-2*****inhibitable allele**	https://www.ncbi.nlm.nih.gov/geo/query/acc.cgi?acc=GSE46912
**MAK-2 phosphopeptide targets study 1**	https://journals.plos.org/plosgenetics/article?id=10.1371/journal.pgen.1004783#s5
**MAK-2 phosphopeptide targets study 2**	https://www.genetics.org/content/203/1/319.supplemental
**Microarray analysis of a Δ*****hog1*****mutant in*****Cryptococcus neoformans***	https://www.ncbi.nlm.nih.gov/geo/query/acc.cgi?acc=GSE16692
**RNA-seq experiment using an*****Aspergillus fumigatus*****Δ*****sakA*****mutant**	https://onlinelibrary.wiley.com/doi/full/10.1111/cmi.12681

## Abbreviations

FGSC: Fungal Genetics Stock Center
FAMD: Factor Analysis of Mixed Data
Ward’s: Ward’s minimal variance
PAM: Partitioning around Medoids
HAC: Hierarchical Agglomerative Clustering
GPCR: G-Protein Coupled Receptor
FunCat: Functional Category
MAPK: Mitogen-Activated Protein Kinase
ERK: Extracellular-signal Regulated Kinase
ROS: Reactive Oxygen Species
STKinase: Serine-Threonine Kinase
Protein Pase: tyrosine protein kinase
