# Supplementary material for: Clustering analysis of large-scale phenotypic data in the model filamentous fungus Neurospora crassa
Source: BMC Genomics. 2020 Nov 2;21:755. doi: 10.1186/s12864-020-07131-7 (PMC7607824; doi:10.1186/s12864-020-07131-7)
Supplement: Supplementary file 2 — Additional file 2. [file 12864_2020_7131_MOESM2_ESM.pdf]

Additional File 2: Scripts used in this study

```


#Pearson's coefficient correlation with complete hierarchical agglomerative clustering

library(factoextra)


setwd('YOUR_WORKING_DIRECTORY/FOLDER')

inData <- "FILENAME"
df <- data.frame(read.csv(paste0(inData,".csv"),
                             header=T,
                             row.names=1,
                             check.names=FALSE))

df_scaled <- scale(df, center = T, scale = T)


dis.cor <- get_dist(df_scaled, method = 'pearson')

clusters <- hclust(dis.cor, method ='complete')

i=2
while (i < 41){
   clust <- cutree(clusters, k=i)
   clust <- as.data.frame(clust)
   df$cluster <- clust$clust

   write.csv(df,paste0('Pearsons_',i,'_clusters.csv'))
   print(i)
   i = i+1
}

#weighting the variables
t_df_scaled <- t(df_scaled)

weighted_corr <- cov.wt(t_df_scaled, wt = c(6,6,1,1,1,1), cor = T)
corr.matrix <- weighted_corr$cor

corr.dis <- dist(corr.matrix)

corr.data <- hclust(corr.dis, method = 'complete')

i=2
while (i < 41){
   clust <- cutree(corr.data, k=i)
   clust <- as.data.frame(clust)
   df$cluster <- clust$clust

   write.csv(df,paste0('Weighted_ pearsons_',i,'_clusters.csv'))
   print(i)
   i = i+1
}
```

```
#K-means clustering

library(FactoMineR)
library(factoextra)
library(cluster)

setwd('YOUR_WORKING_DIRECTORY/FOLDER')
inData <- 'FILENAME'

data <- read.csv(paste0(inData,'.csv'),
                 header=T,
                 sep=",",
                 row.names=1,
                 check.names=FALSE)
data <- as.matrix(data)
i=2

while (i< 50) {
kdata <- kmeans(data, center = i, nstart=25)

gap_stat <- clusGap(data, FUN=kmeans, nstart =25, K.max=i, B=50)
print(i)

distance <- get_dist(data)

#creates a pdf of several plots
pdfname <- paste0('K-means',i,'clusters.pdf')
pdf(pdfname,
    width = 11,
    height = 11)

fviz_nbclust(data, kmeans, method='wss', k.max=i)
fviz_nbclust(data, kmeans, method='silhouette', k.max=i)
fviz_gap_stat(gap_stat)
fviz_dist(distance)
fviz_cluster(kdata, data=data)

dev.off()

#creates spreadsheet of input data with cluster assignments
attach_Data$cluster <- kdata$cluster
csvname <- paste0('K-means',i,'clusters.csv')
write.csv(attach_Data, csvname)
i = i+1
}
```

```r
#Hierarchical clustering on principle components

library(FactoMineR)
library(factoextra)

setwd('YOUR_WORKING_DIRECTORY/FOLDER')

inData <- 'FILENAME'

data <- read.csv(file=paste0(inData,'.csv'),
                                header=T,
                                sep=',',
                                row.names=1,
                                check.names=FALSE)
data <- as.matrix(data)

just_data <- data[,6:15]
i=2
while (i< 41){
pca <- FAMD(data)
res.hcpc <- HCPC(pca,-1,iter.max = 50, min= i, max =i, graph =T)

#creates spreadsheet of input data with cluster assignments
csvname <- paste0('HCPC clustering ',i,'clusters.csv')
write.csv(res.hcpc$data.clust,csvname)
print(i)
i=i+1
}
```

```r
#Partitioning around medoids
library(cluster)

setwd('YOUR_WORKING_DIRECTORY/FOLDER')
#load data
inData <-
dat =read.csv("KO_Phenotypes_ALL_10_7_28_19_FINAL.csv")
#subset  out measurements
dat1 = dat[,13:18]

#load library to creat dissimilariy matrix with mixed data types (gowers)
library(cluster)

#Different weights for variables
weight0 <- c(1,1,1,1,1,1,1,1,1,1)
weight1 <- c(2,2,1,1,1,1,1,1,1,1)
weight2 <- c(2,2,1,.5,1,.5,1,.5,1,.5,1,.5)
weight3 <- c(6,2,2,2,1,1,1,1,1,1)
weight4 <- c(6,5,.5,.5,1,1,1,1,1,1,1)
weight5 <- c(6,4,1,1,1,1,1,1,1,1)
weight6 <- c(6,6,1,1,1,1,1,1,1,1)
#calculate gower's distance
distF = daisy (dat1, metric = 'gower', weight = weight0)

#Partitioning around medoids
i <-  2
while (i<51)  {
pamfit = pam(distF, diss=TRUE, k=i)
#obtain clusters
clust = pamfit$clustering
## add clusters to data
dat$cluster = clust
#export clusters
write.csv(dat, file = paste0('PAM_',i,'_clusters.csv'))
i = i+1
}

#Ward's minimum variance
hc_fun = hclust(distF, method="ward.D")

i=2
while (i<51){
  clusters <- cutree(hc_fun, k=i)
  data$cluster <- clusters
  write.csv(data, paste0('Ward_',i,'_clusters.csv'))
  print(i)
  i=i+1
}
```

```r
#gets the number per cluster
library(plyr)

setwd('YOUR_WORKING_DIRECTORY/FOLDER')

i=2
statlist <- list()

while (i<41) {
  name <- paste0('FILENAME',i,'.csv')
  data1 <- read.csv(name)
  data2 <- data1$ANY_COLUMN
  Number <- as.data.frame(data2)
  Number$cluster <- sheet$clust

  number <- ddply(Number, ('cluster'), summarise,
                  N = length(data2))

  statlist[[i-1]] <- number$Number

  print(i)
  i=i+1
}

COUNTED <- as.data.frame(t(ldply(statlist, rbind)))
write.csv(COUNTED, 'NEW CSV FILENAME.csv')
```

```
#Binning genes into categories per cluster
library(plyr)
library(dplyr)
library(cowplot)

filename <- 'FILENAME.csv'
data <- read.csv(data)

#Group the data by a factor (column)
data$group1 <- cut('Grouping factor1', c(0,40,65,75,77.5,82.5,85,100)) #Grouping factor can be any column from data, numeric vector (c(...)) is provided as an example
data$group2 <- cut('Grouping factor2', c(0,15,25,30,35,40,45,100))

#Converts the numeric cuts into categories
levels(data$group1) <- c('Severly Reduced','Reduced','Slightly Reduced','Normal Low', 'Normal Average', 'Normal High', 'Increased')
levels(data$group2) <- c('Severly Reduced','Reduced','Slightly Reduced','Normal Low', 'Normal Average', 'Normal High', 'Increased')

supersheet <- data[,(ncol(data)-2):(ncol(data))]

sum <- supersheet %>%
  group_by(cluster, group) %>%
  summarise(number = length(group)) %>%
  mutate(percent = (number / sum(number))*100)

sumah <- supersheet %>%
  group_by(cluster, ahgroup) %>%
  summarise(ahnumber = length(ahgroup)) %>%
  mutate(percent = (ahnumber / sum(ahnumber))*100)

#Plots
Growth <- ggplot(sum, aes(x=cluster, y = percent, fill = group))+
  geom_bar(stat ='identity')+
  geom_text(data = subset(sum,percent !=0),aes(label=paste0(round(percent), "%")),
            position = position_stack(vjust = 0.5), size =4, fontface='bold')+
  geom_text(data = sum, aes(label=number), position = position_stack(vjust=0.4), size = 3, fontface='bold')+
  theme(axis.text.x = element_text(angle = 65, hjust = 1, face='bold', color='black', size=20),
        axis.text.y = element_text(face='bold', color='black', size=20),
        axis.title = element_text(face='bold', color='black', size=20 ),
        plot.title = element_text(face='bold', color='black', size=20))

AH <- ggplot(sumah, aes(x=cluster, y = percent, fill = ahgroup))+
  geom_bar(stat ='identity')+
  geom_text(data = subset(sumah,percent !=0),aes(label=paste0(round(percent), "%")),
            position = position_stack(vjust = 0.5), size =4, fontface='bold')+
  geom_text(data = sumah, aes(label=ahnumber), position = position_stack(vjust=0.4), size = 3, fontface='bold')+
  theme(axis.text.x = element_text(angle = 65, hjust = 1, face='bold', color='black', size=20),
        axis.text.y = element_text(face='bold', color='black', size=20),
        axis.title = element_text(face='bold', color='black', size=20 ),
        plot.title = element_text(face='bold', color='black', size=20))


summary <- merge(sum, sumah, by='cluster')

write.csv(summary, 'PAM 0 20 Growth and AH summary.csv')

pdf('Binned groups by cluster PAM 0 20.pdf', height = 12, width = 36)
plot_grid(Growth, AH, labels =c('A.', 'B.'), nrow = 1)
dev.off()
```

```r
#Average consensus and average relative standard deviation

library(plyr)
library(dplyr)
library(tidyr)
library(purrr)

################################FUNCTIONS####################################################
count_words <- function(data, column, clustercol) {
  datacolumn <- data[,column]
  datacolumn <- as.data.frame(datacolumn)
  datacolumn$cluster <- data[,clustercol]

  df <- datacolumn %>%
    group_by(cluster)%>%
    mutate(words= strsplit(as.character(datacolumn),' '))%>%
    unnest()%>%
    dplyr::count(cluster, words) %>%
    transmute(n, pcnt = (n/sum(n)*100))

  df <- ddply(df, c('cluster'), summarise, value = max(pcnt))
    max_pcnt_average <- mean(df$value)
  return(max_pcnt_average)
}

relative_standard_deviations <- function(data, column, clustercol) {
    df <- data[,column]
    df <- as.data.frame(df)
    df$cluster <- data[,clustercol]

    summary_data <- ddply(df, c('cluster'), summarise,
                          N = length(df),
                          mean = mean(df),
                          sd = sd(df),
                          relative_sd = ((sd/mean)*100))
    relative_sd_means <- colMeans(summary_data[,5,drop=FALSE])
  return(relative_sd_means)
}
##############################################################################################

setwd('YOUR_WORKING_DIRECTORY/FOLDER')

#Empty lists where information will be stored
rgsdlist <- list()
rahsdlist <- list()
rwordlist <- list()
rfinalwordlist <- list()

#The main loop that calculates the relative standard deviation of phenotypic traits per cluster
i= 20
while (i<34) {
    name <- paste0('FILENAME_',i,'_clusters.csv')

    sheet <- read.csv(name, row.names = 1)
    sheet <- sheet[,9:19] #choose columns that need to be analyzed

    rsdmeans <- relative_standard_deviations(sheet, 1, 11)
    sdname <- paste('Gsd',i)
    rgsdlist[[sdname]] <- rsdmeans #adds the relative standard deviation for one continous variable to a list

    rsdmeans2 <- relative_standard_deviations(sheet, 2, 11)
    sdname <- paste('AHsd',i)
    rahsdlist[[sdname]] <- rsdmeans2 #adds the relative standard deviation for one continous variable to a list

```

```
    #iterates through the columns with categorical data
    word_clusters <- paste0('cluster',i)
    k=3
    while (k < 11){
        rwordlist[[k-2]] <- count_words(sheet, k, 11)
        rfinalwordlist[[word_clusters]] <- rwordlist
        k=k+1
    }
    print(i)
    i=i+1
}

#Binds the data stored in lists to a datafram and then outputs a .csv file
do.call('rbind', rfinalwordlist)
relative_sd_frame <- data.table::rbindlist(rfinalwordlist)
names(relative_sd_frame) <- c('C num', 'C morph', 'PP num', 'PP morph', 'P num', 'P morph', 'A num', 'A morph') #names the columns
relative_sd_frame$growth <- unlist(rgsdlist)
relative_sd_frame$AH     <- unlist(rahsdlist)

write.csv(relative_sd_frame, 'Summary_info_PAM.csv')
```

```
#K-means clustering of expression data

my_scale <- function(x) { #scales the data between -2 and 2
  m = apply(x, 1, mean, na.rm = T)
  s = apply(x, 1, sd, na.rm = T)
  return((x - m) / s)
}

wang <- factor(c(0,2,24,48,72,96,120,144))
wangnum <- c('0 h', '2 h', '24 h', '48 h', '72 h', '96 h', '120 h', '144 h')
germ <- factor(c(0.25,2,4,6))
germnum <- c('1/4 h', '2 h', '4 h', '6 h')
green <- factor(c(0,2,4,8,10,12,12.2,14,18,24))
greennum <- c('0 h', '2 h', '4 h','8 h', '10 h','12B','12T', '14 h', '18 h','24 h')
wanggerm <- factor(c(0.25, 2, 4, 6))
wanggermnum <- c('0.25 h','2 h','4 h' ,'6 h')
kasuga <- factor(c(1,3,9,15,21,27))
kasuganum <- c('0 hr', '3 hrs', '9 hrs', '15 hrs', '21 hrs', '27 hrs')

library(ggplot2)
library(ggthemes)
library(lemon)
library(cluster)
library(factoextra)

setwd('YOUR_WORKING_FOLDER')

inData <- 'FILENAME'
i <- 7 #number of clusters

df <- data.frame(read.csv(file=paste(inData,".csv",sep=""),
                           header=T,
                           row.names=1,
                           check.names=FALSE))
clean_df <- df[1:10] #colums with data for scaling
cleaner_df <- my_scale(clean_df)

fit <- kmeans(cleaner_df, i, nstart = 50)
output <- data.frame(cleaner_df, fit$cluster)
output <- as.data.frame(output)

graph <- tibble::rownames_to_column(output, 'gene')
cluster_graph <- tidyr::gather(graph, key, value, -fit.cluster, -gene)
uh <- cluster_graph$key
uh <- gsub("[a-zA-Z]", "", uh)
uh <- as.numeric(uh)
cluster_graph$key <- uh
cluster_graph$key <- as.factor(cluster_graph$key)
cluster_graph <- as.data.frame(cluster_graph)
cluster_graph$TF <- df$TF

tiff(paste0('k-means green clustering ',i,' clusters.tiff'),
     width=3840,
     height=2160,
     units='px')

ggplot(cluster_graph, aes(key, value, group = gene, ))+
  geom_point(aes(color = factor(fit.cluster)))+
  geom_line(aes(color = factor(fit.cluster)))+
  facet_rep_wrap(~fit.cluster, repeat.tick.labels='all')+
  theme_tufte()+
  theme(axis.line = element_line(), legend.position = 'none',
              axis.text.x = element_text(angle = 65, hjust = 1, face='bold',
              color='black', size=40),
              axis.text.y = element_text(face='bold', color='black', size=40),
              axis.title = element_text(face='bold', color='black', size=40),
              strip.text = element_text(face='bold',size=40))+
  scale_color_manual(breaks =cluster_graph$fit.cluster, values = c('#e6194b',
  '#3cb44b', '#ffe119', '#4363d8', '#f58231', '#911eb4',
```

```r
                         '#46f0f0', '#f032e6', '#bcf60c', '#fabebe', '#008080', '#e6beff',
                         '#9a6324', '#fffac8', '#800000', '#aaffc3', '#808000', '#ffd8b1',
                         '#000075', '#808080', '#ffffff', '#000000'))+
  scale_x_discrete(limits = green,
                   labels = greennum)


dev.off()

output$TF <- df$TF
write.csv(output, paste0('K-means green ',i,' clusters with TF.csv'))
```
