# Supplementary material for: Clustering analysis of large-scale phenotypic data in the model filamentous fungus Neurospora crassa
Source: BMC Genomics. 2020 Nov 2;21:755. doi: 10.1186/s12864-020-07131-7 (PMC7607824; doi:10.1186/s12864-020-07131-7)
Supplement: Supplementary file 5 — Additional file 5. [file 12864_2020_7131_MOESM5_ESM.docx]

**Additional File 5**

**Clustering methods comparison**

We tested several algorithms and methods of clustering data. First, we tested the method most often used in previous studies with fungi (1-3) by creating a distance matrix with One Minus Pearson’s correlation coefficient (4) and then clustering the genes using Hierarchical Agglomerative Clustering (HAC) (5). Since Pearson’s Correlation Coefficient cannot use categorical data to create the distance matrix, we converted the categorical data to numerical values. All ordinal categories were converted to the same scale, a value between 0-1.5 based on the severity of the phenotype. The conversion could only be performed with the semi-quantitative categorical variables (ordinal categories): conidia, protoperithecia, perithecia, and ascospore abundances, for a total of six phenotypes. As per our criteria above, we examined a range of 17 to 19 clusters in detail (Additional File 3). Analysis of the relative standard deviations for each clustering run revealed that basal hyphae growth rate and aerial hyphae height had high relative standard deviations, with an average of 26.07 and 32.22, respectively (Additional File 4). The average percent consensus for the categorical phenotypes was fairly high, with the average for all phenotypes being 85.7% (Additional File 4). We tried weighting basal hyphae growth rate and aerial hyphae height six times higher than the other phenotypes, in an attempt to lower the relative standard deviation of these two phenotypes. With this increased weight for the two phenotypes, the range of clusters that fell within our criteria shifted to 18-28 (Additional File 3) and the relative standard deviations in growth rate and aerial hyphae height decreased to an average of 19.29 and 26.79, respectively (Additional File 4). At the same time, the average percent consensus of the ordinal traits lowered modestly to 81.4% (Additional File 3).

Our second approach implemented a partitioning method to cluster the phenotypic data. One of the most well-known such methods is K-means (6). As with the One Minus Pearson’s Correlation Coefficient distance matrix, K-means cannot process categorical data. Therefore, we used the same converted data set described above for the K-means clustering runs. The range of clusters examined was relatively low, from 20 to 23 clusters (Additional File 3). The average standard deviations for both basal hyphae growth rate (23.79) and aerial hyphae height (22.86) were lower than those obtained using non-weighted HAC. The average percent consensus for all ordinal traits was 92.5%, higher than non-weighted (85.7%) and weighted HAC (81.4%) (Additional File 4). While performing better that HAC, K-means still yielded high relative standard deviations for basal hyphae growth rate and aerial hyphae height.

Based on the unsatisfactory results using the converted dataset, we turned to methods that would require little or no pre-processing of our data and that would retain the categorical data. An algorithm that can handle such mixed data is FAMD (7). However, this approach quickly failed our criteria, as the run with three total clusters contained one cluster with a single gene (Additional File 3). Additionally, k-prototypes ([**https://doi.org/10.1023/A:1009769707641**](https://doi.org/10.1023/A:1009769707641)) was tried and found to be unstable and multiple runs would not converge on similar numbers of clusters. We utilized the Daisy function (5) and Gower’s metric (8) from the “cluster” R package to create a dissimilarity matrix (r-project.org). We then used Ward’s (9) or PAM (10) algorithms to generate clusters. The range of runs examined was 18-22 for Ward’s and 18-21 for PAM. Both PAM and Ward’s clustering resulted in similar, large relative standard deviations for growth rate (PAM: 26.25; Ward’s: 26.95) and aerial hyphae height (PAM: 32.10; Ward’s: 33.48) (Table 1, Additional File 4). However, the clustering of categorical phenotypes using PAM was much more consistent than what was observed with HAC, K-means, FAMD and Ward’s (HAC: 85.7%; K-means: 92.5%; Ward’s: 93.4%; PAM: 95.4%) (Additional File 4). To reduce the magnitude of the ranges and standard deviations of the two continuous phenotypes, we varied the weights of specific phenotypes during the clustering using PAM and Ward’s (Table 1, Additional File 3, Additional File 4). We selected the analysis using a weighted PAM with 40 clusters as it had the lowest average relative standard deviation for the continuous phenotypes and the highest average percent consensus for the categorical phenotypes (Table 1). This approach weighted basal hyphae growth rate and aerial hyphae height six times greater than all other phenotypes. The final average standard deviations for basal hyphae growth rate and aerial hyphae height were 13.94 and 11.86, respectively, with all categorical data at an average of 94.15% coverage, with a standard deviation of 10.5 (Table 1).

**References**

1. Brown JA, Sherlock G, Myers CL, Burrows NM, Deng C, Wu HI, et al. Global analysis of gene function in yeast by quantitative phenotypic profiling. Mol Syst Biol. 2006;2:2006 0001.

2. Lee KT, So YS, Yang DH, Jung KW, Choi J, Lee DG, et al. Systematic functional analysis of kinases in the fungal pathogen *Cryptococcus neoformans*. Nat Commun. 2016;7:12766.

3. Son H, Seo YS, Min K, Park AR, Lee J, Jin JM, et al. A phenome-based functional analysis of transcription factors in the cereal head blight fungus, *Fusarium graminearum*. PLoS Pathog. 2011;7(10):e1002310.

4. Freedman D, Pisani R, Purves R. Statistics. 4th ed. Pisani R, Purves R, editors: WW Norton & Company, New York, NY; 2007.

5. Maechler M, Rousseeuw P, Struyf A, Hubert M, Hornik K. Cluster: Cluster Analysis Basics and Extensions. R package version 2.1.0. . 2019.

6. Hartigan JA, Wong MA. Algorithm AS 136: A K-means clustering algorithm. Applied Statistics. 1979;28:100-8.

7. Audigier V, Husson F, Josse J. A principal component method to impute missing values for mixed data. Adv Data Anal Classif. 2016;10:5.

8. Gower JC. A General Coefficient of Similarity and Some of Its Properties. Biometrics. 1971;27:857-71.

9. Schubert E, Rousseeuw PJ. Faster k-Medoids Clustering: Improving the PAM, CLARA, and CLARANS Algorithms. Machine Learning2019.

10. Szekely G, Rizzo M. Hierarchical Clustering via Joint Between-Within Distances: Extending Ward's Minimum Variance Method. Journal of Classification. 2005;22:151.
