# Supplementary material for: Clustering analysis of large-scale phenotypic data in the model filamentous fungus Neurospora crassa
Source: BMC Genomics. 2020 Nov 2;21:755. doi: 10.1186/s12864-020-07131-7 (PMC7607824; doi:10.1186/s12864-020-07131-7)
Supplement: Supplementary file 7 — Additional file 7. [file 12864_2020_7131_MOESM7_ESM.pdf]

Additional File 7

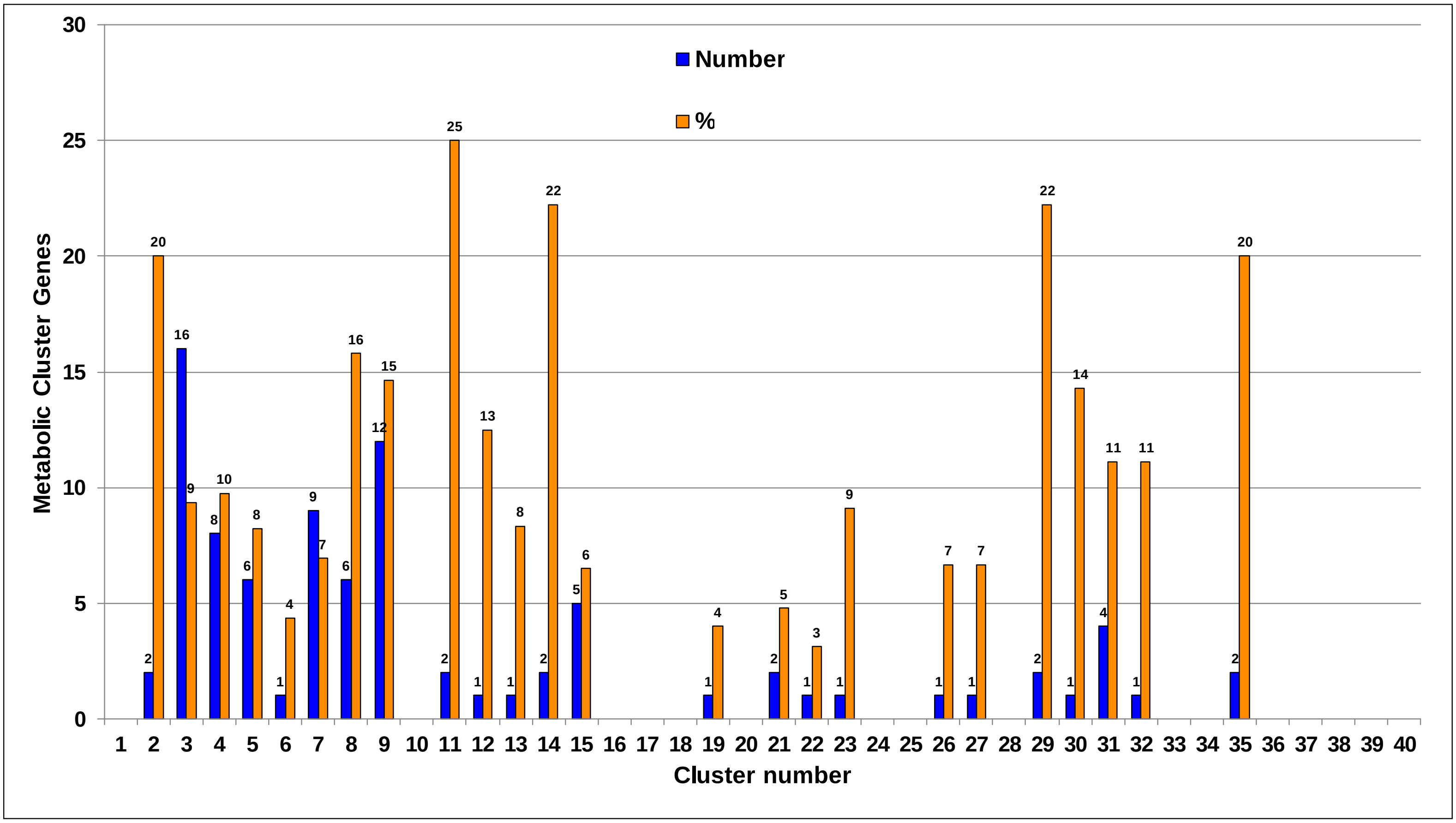

Distribution of metabolic genes in clusters. The number of metabolic genes in each cluster, along with the % representation in each cluster is shown (actual numbers on top of bars). The list of metabolic genes is from Dreyfuss et al., 2013.

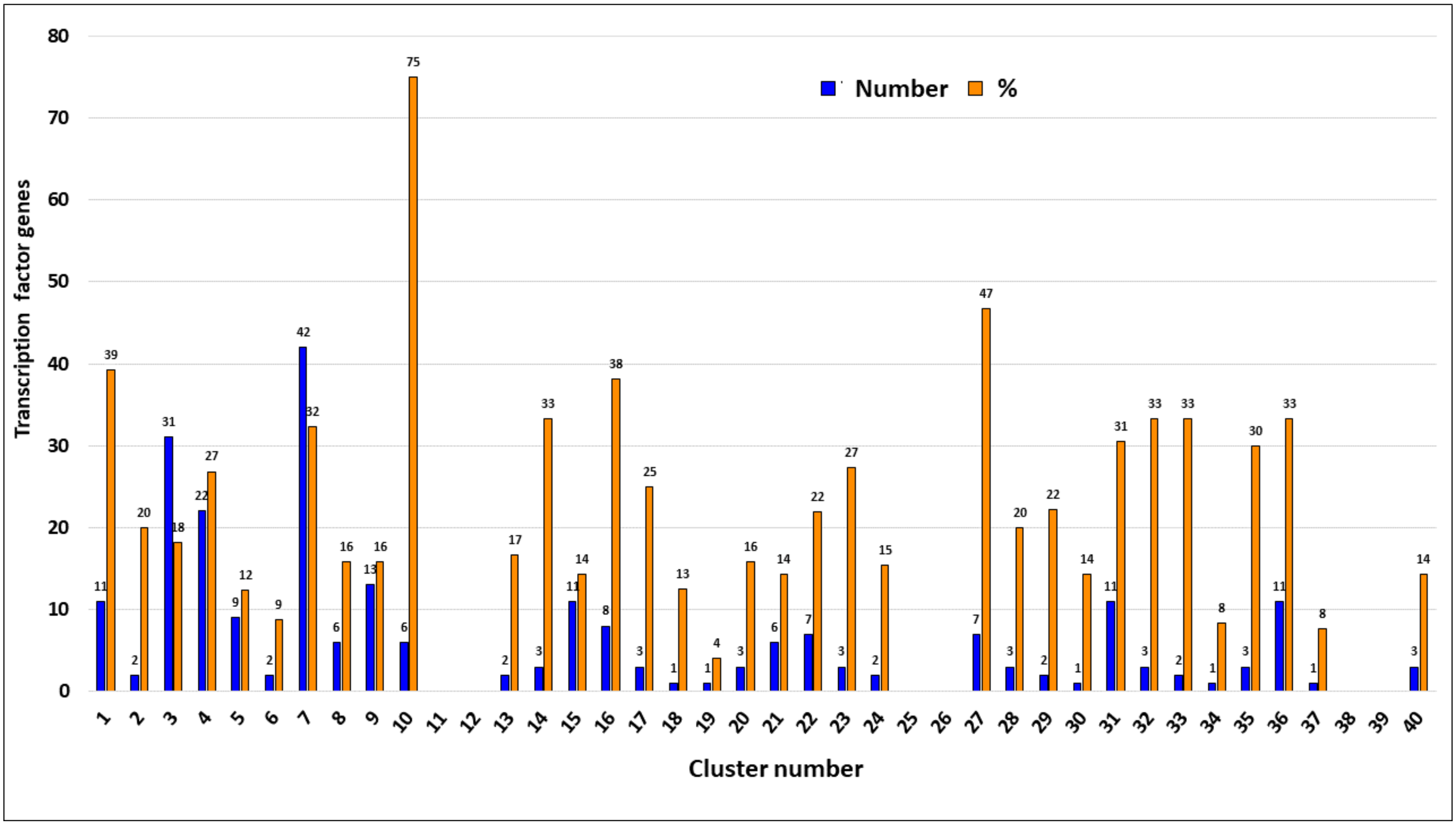

Distribution of transcription factors in clusters. The number of genes encoding a transcription factor in each cluster, along with the % representation in each cluster is shown (actual numbers on top of bars). The list of transcription factor genes is from Carrillo et. al., 2015.
