# Supplementary material for: Clustering analysis of large-scale phenotypic data in the model filamentous fungus Neurospora crassa
Source: BMC Genomics. 2020 Nov 2;21:755. doi: 10.1186/s12864-020-07131-7 (PMC7607824; doi:10.1186/s12864-020-07131-7)
Supplement: Supplementary file 9 — Additional file 9. [file 12864_2020_7131_MOESM9_ESM.pdf]

Additional File 9

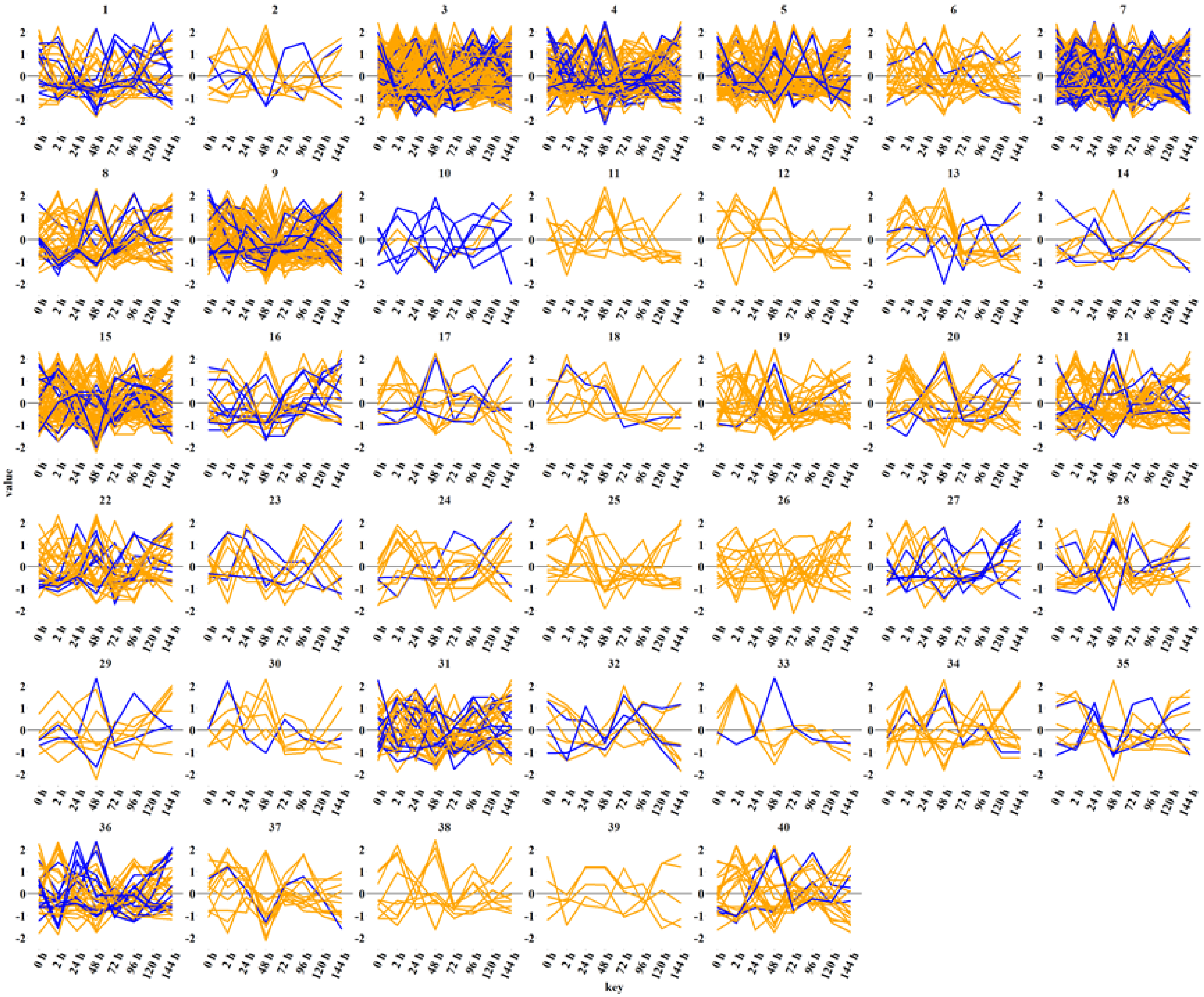

Expression of cluster genes during a sexual development time course. RNAseq data were obtained from Wang et al., 2014. The expression value for each gene was standardized to have a mean of 0 and a standard deviation of 2 across the eight time points in the dataset. Each gene is represented as a single line from 0 h to 144 h, with the blue lines representing transcription factors and all other genes as orange.

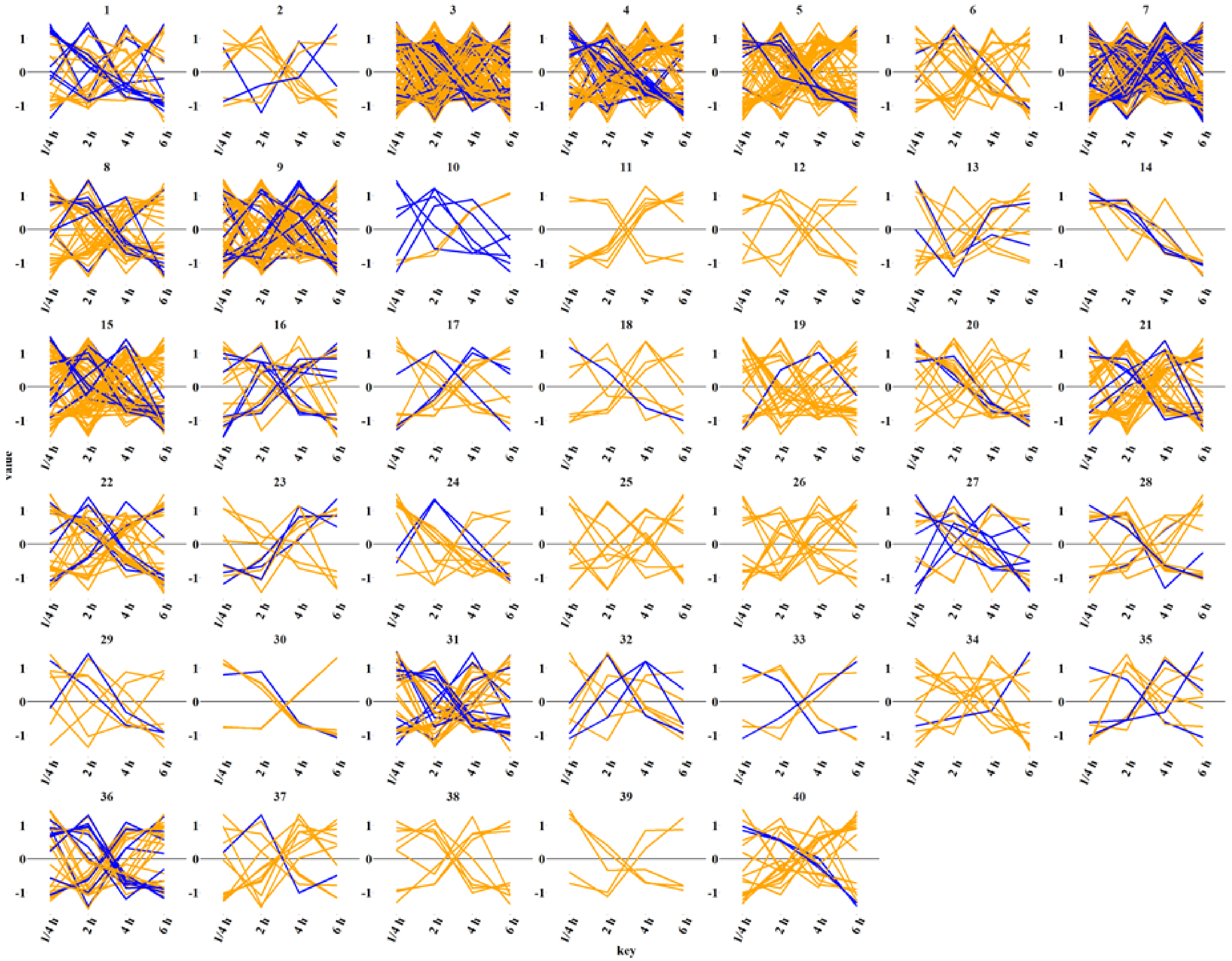

Supplemental figure 4. Expression of genes during a conidial germination timecourse per cluster. RNAseq data were obtained from Wang et. al., 2019. The expression value for each gene was standardized to have a mean of 0 and a standard deviation of 2 across the four samples. Each gene is represented as a single line from 0.25 h to 6 h, with the blue lines representing transcription factors and all other genes as orange.

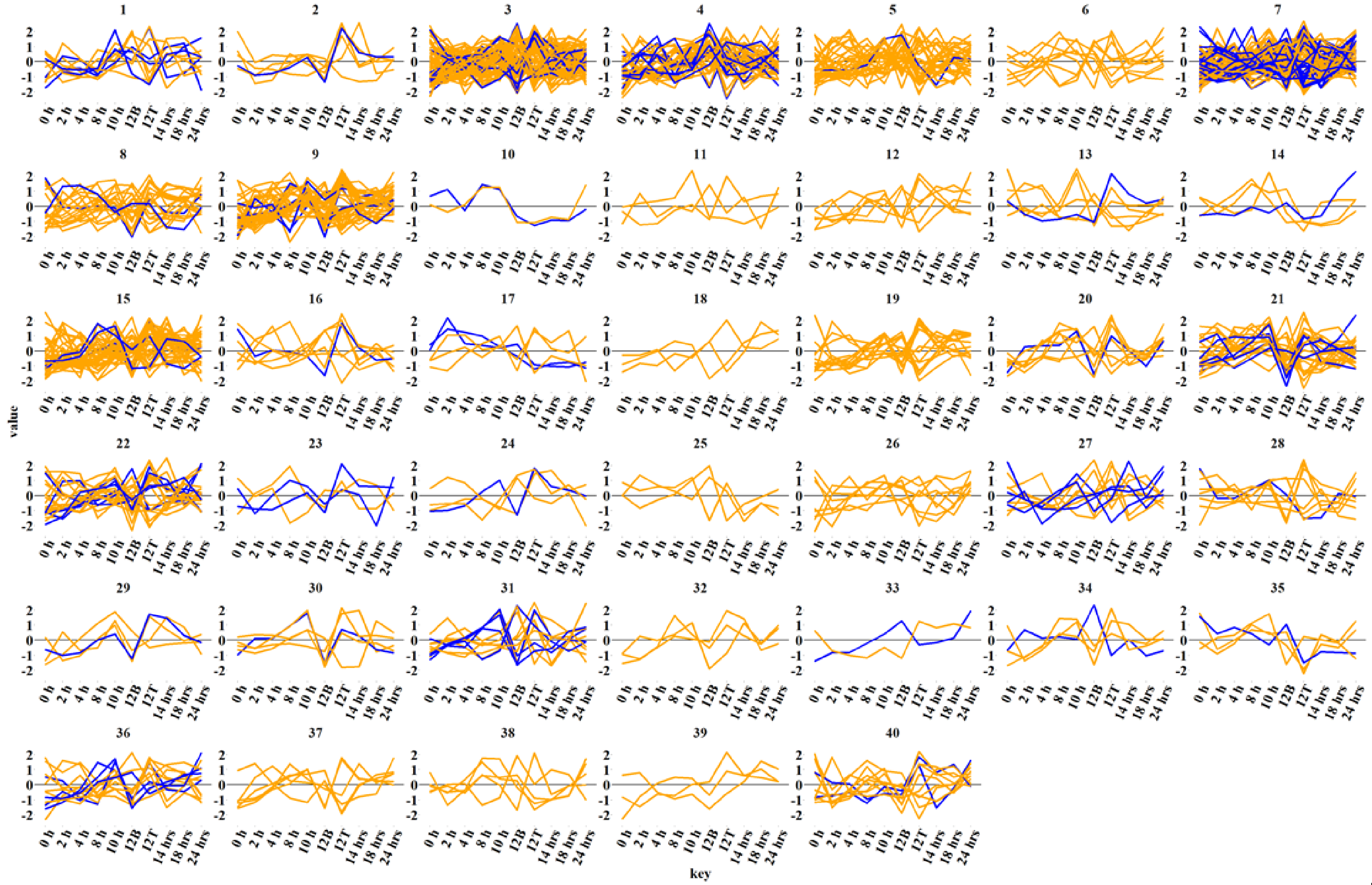

Expression of genes during a conidiation timecourse per cluster. Microarray data were obtained from Greenwald et. al., 2010. The expression value for each gene was standardized to have a mean of 0 and a standard deviation of 2 across the 10 samples. Each gene is represented as a single line from 0 h to 24 h, with the blue lines representing transcription factors and all other genes as orange.

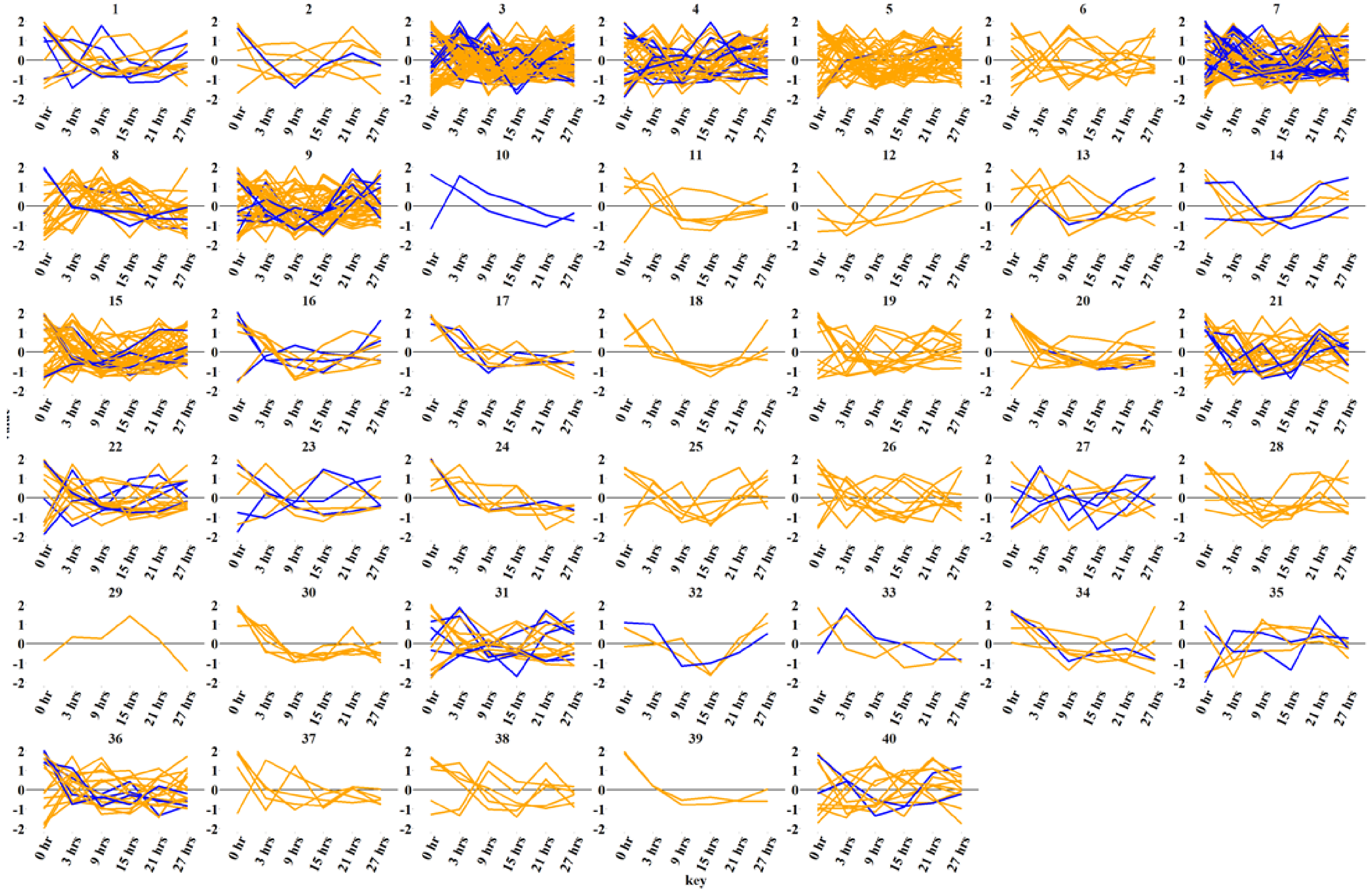

Expression of genes during colony a development timecourse per cluster. Microarray data were obtained from Kasuga et. al., 2008. The expression value for each gene was standardized to have a mean of 0 and a standard deviation of 2 across the six samples. Each gene is represented as a single line from 0 h to 27 h, with the blue lines representing transcription factors and all other genes as orange.

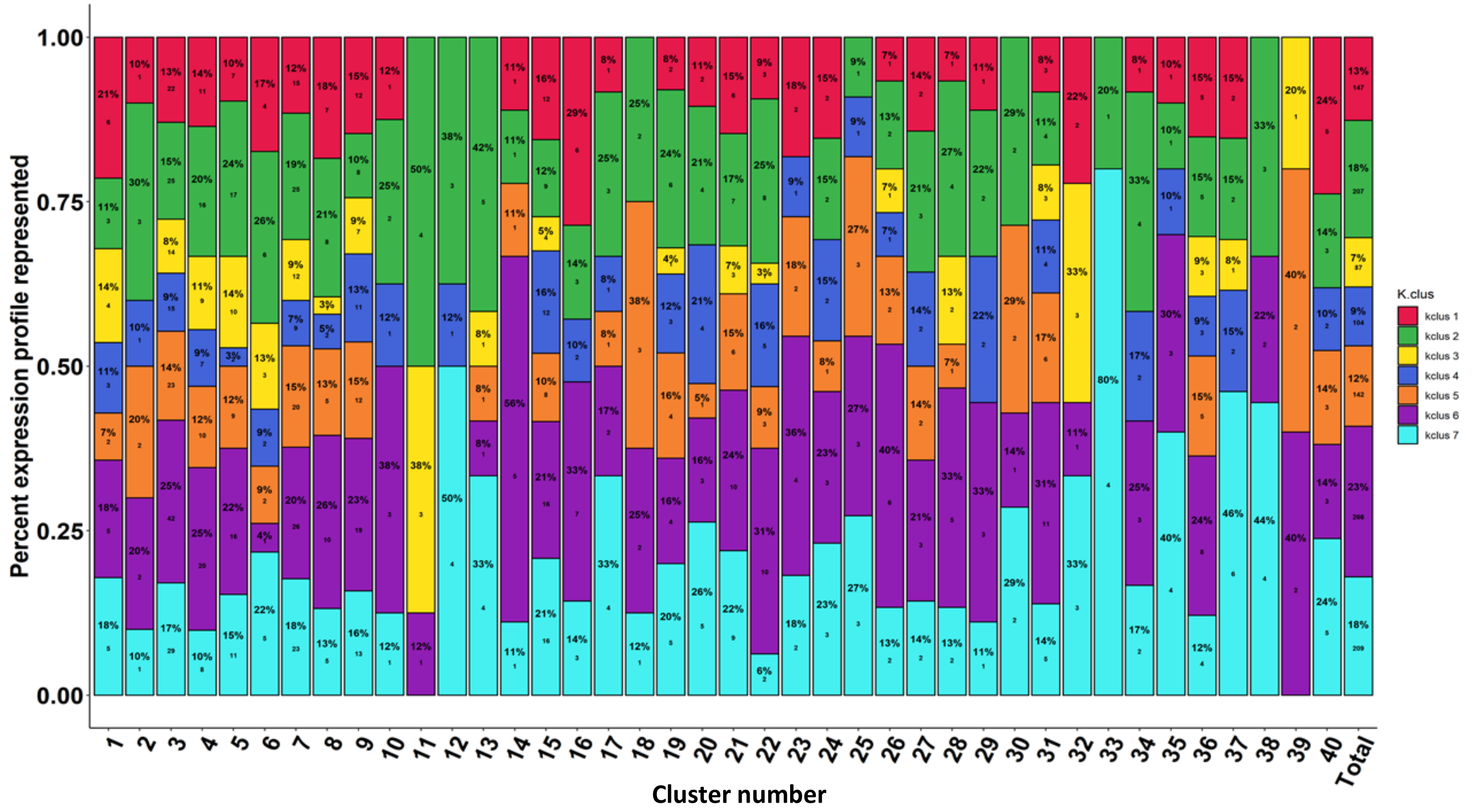

Distribution of expression profiles in each cluster during the sexual development time course. RNAseq data were obtained from Wang et. al., 2014. The expression value of each gene was standardized to have a mean of 0 and a standard deviation of 2 across the eight samples. Genes were partitioned into seven expression clusters using K-means clustering. Each colored portion of each bar denotes the percent of genes in that cluster that belong to the corresponding expression profile.

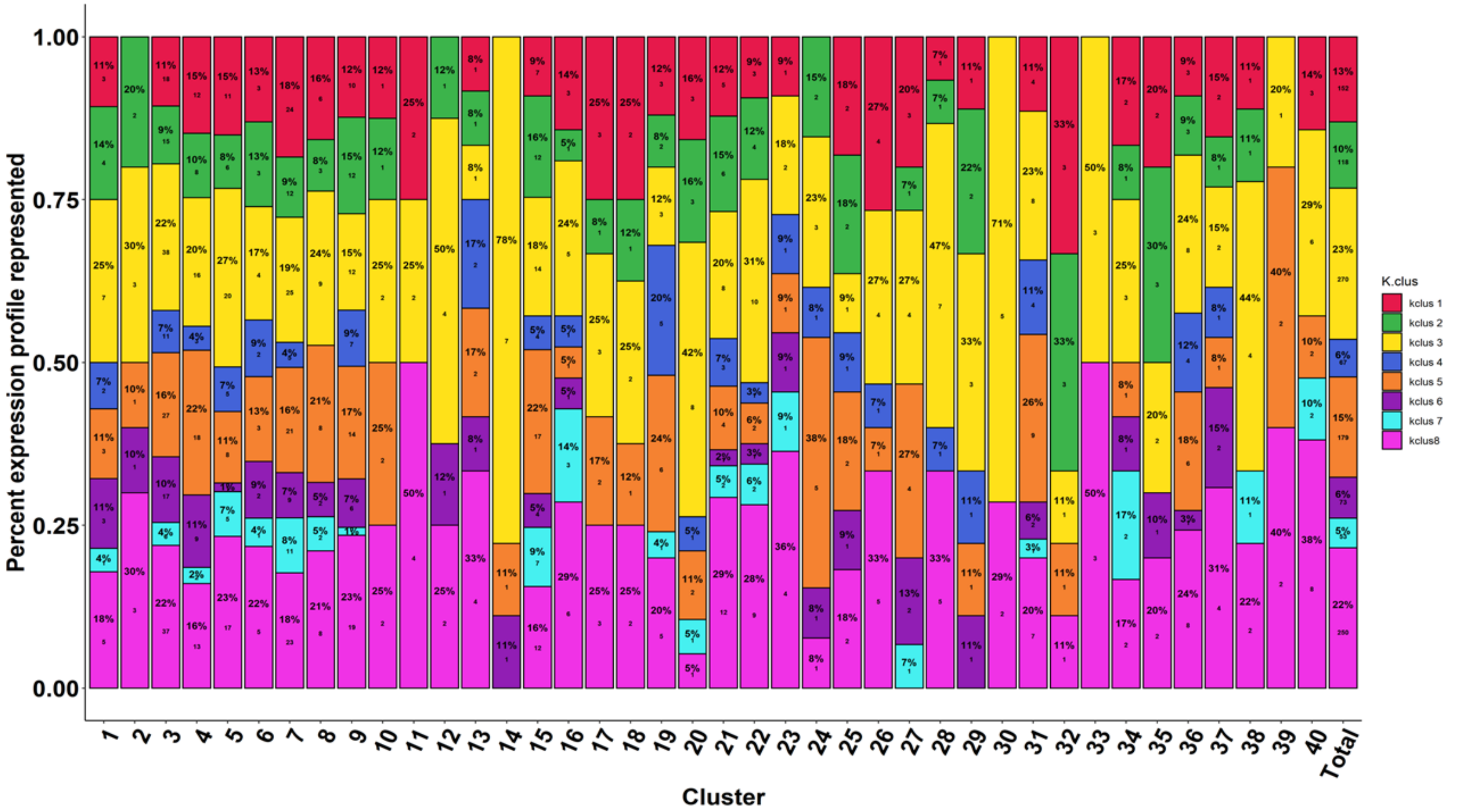

Distribution of expression profiles per cluster during a conidial germination timecourse. RNAseq data were obtained from Wang et. al., 2019. The expression value of each gene was standardized to have a mean of 0 and a standard deviation of 2 across the four samples. Genes were partitioned into eight expression clusters using K-means clustering. Each colored portion of each bar denotes the percent of genes in each cluster that belong to the corresponding expression profile.

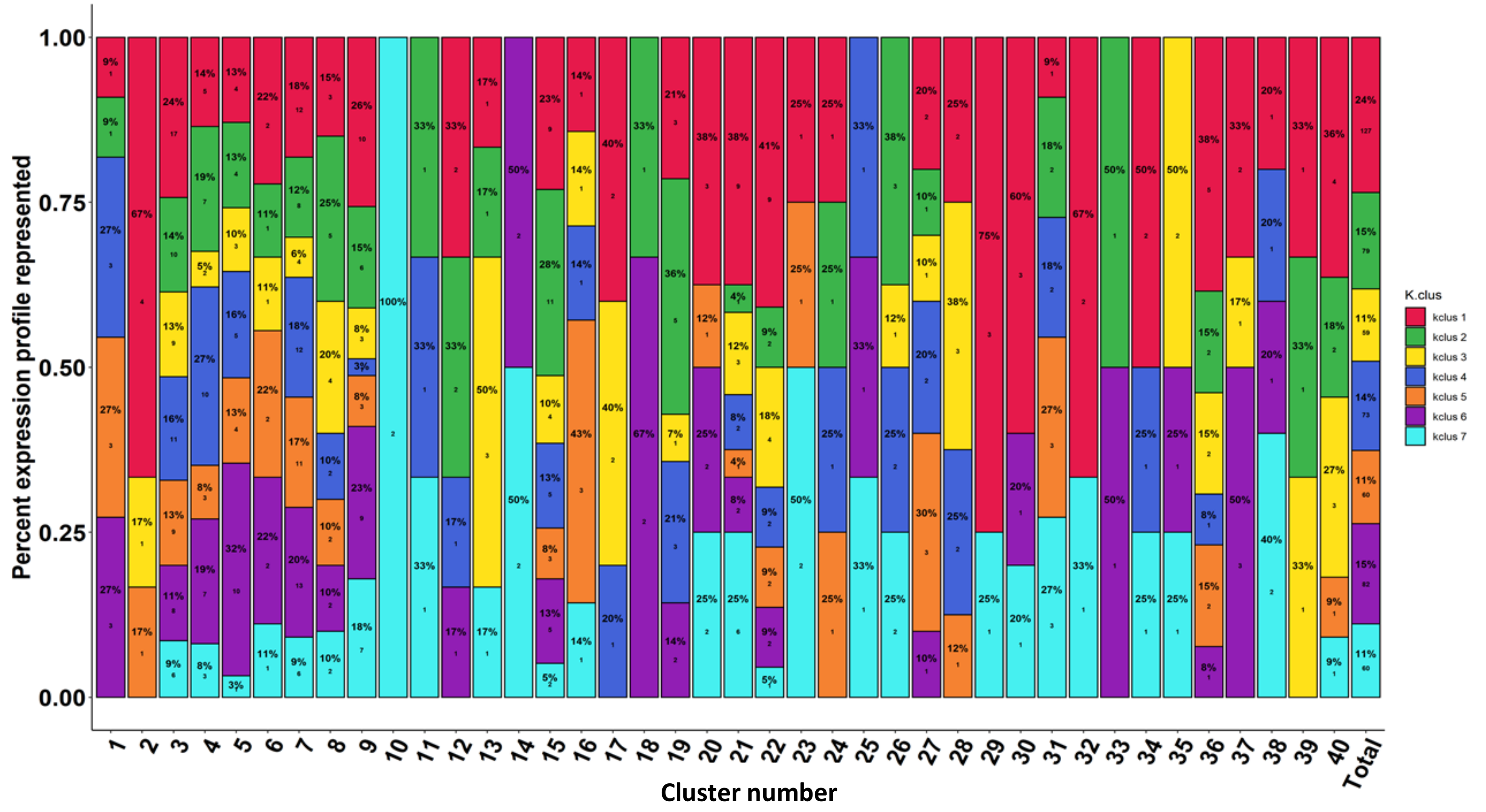

Distribution of expression profiles per cluster during a conidiation timecourse.
Microarray data were obtained from Greenwald et. al., 2010. The expression value of each gene was standardized to have a mean of 0 and a standard deviation of 2 across the 10 samples. Genes were partitioned into seven expression clusters using K-means clustering. Each colored portion of each bar denotes the percent of genes in each cluster that belong to the corresponding expression profile.

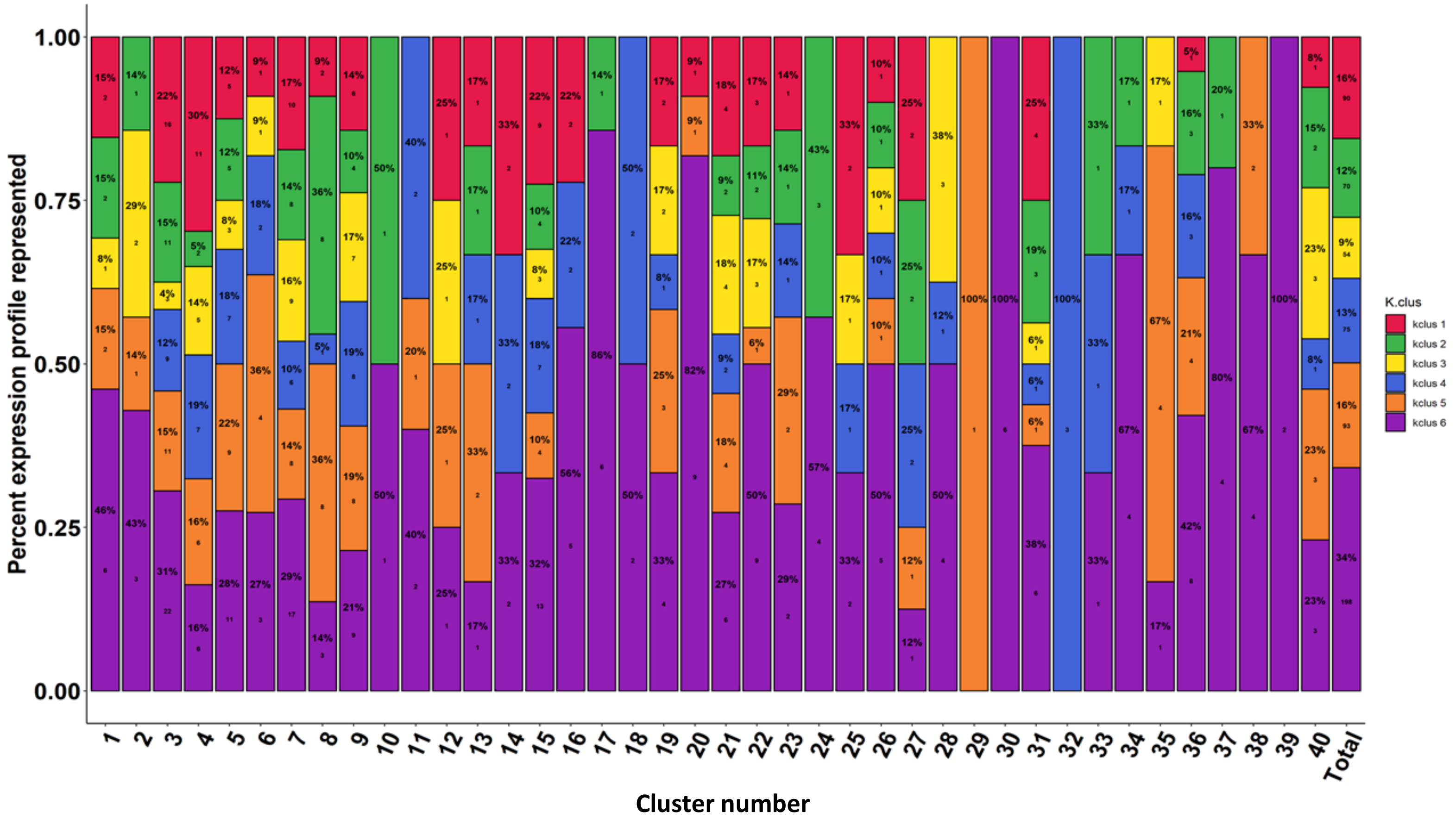

Distribution of expression profiles per cluster during a colony development timecourse. Microarray data were obtained from Kasuga et. al., 2008. The expression value of each gene was standardized to have a mean of 0 and a standard deviation of 2 across the six samples. Genes were partitioned into six expression clusters using K-means clustering. Each colored portion of each bar denotes the percent of genes in each cluster that belong to the corresponding expression profile.
